# A metabolic redox relay supports ER proinsulin export in pancreatic islet **β** cells

**DOI:** 10.1172/jci.insight.178725

**Published:** 2024-06-27

**Authors:** Kristen E. Rohli, Nicole J. Stubbe, Emily M. Walker, Gemma L. Pearson, Scott A. Soleimanpour, Samuel B. Stephens

**Affiliations:** 1 Fraternal Order of Eagles Diabetes Research Center,; 2 Interdisciplinary Graduate Program in Genetics, and; 3 Division of Endocrinology and Metabolism, Department of Internal Medicine, University of Iowa, Iowa City, Iowa, USA.; 4 Division of Metabolism, Endocrinology and Diabetes, Department of Internal Medicine, and; 5 Department of Molecular and Integrative Physiology, University of Michigan, Ann Arbor, Michigan, USA.; 6 VA Ann Arbor Healthcare System, Ann Arbor, Michigan, USA.

**Keywords:** Cell biology, Endocrinology, Beta cells, Insulin, Protein traffic

## Abstract

ER stress and proinsulin misfolding are heralded as contributing factors to β cell dysfunction in type 2 diabetes, yet how ER function becomes compromised is not well understood. Recent data identify altered ER redox homeostasis as a critical mechanism that contributes to insulin granule loss in diabetes. Hyperoxidation of the ER delays proinsulin export and limits the proinsulin supply available for insulin granule formation. In this report, we identified glucose metabolism as a critical determinant in the redox homeostasis of the ER. Using multiple β cell models, we showed that loss of mitochondrial function or inhibition of cellular metabolism elicited ER hyperoxidation and delayed ER proinsulin export. Our data further demonstrated that β cell ER redox homeostasis was supported by the metabolic supply of reductive redox donors. We showed that limiting NADPH and thioredoxin flux delayed ER proinsulin export, whereas thioredoxin-interacting protein suppression restored ER redox and proinsulin trafficking. Taken together, we propose that β cell ER redox homeostasis is buffered by cellular redox donor cycles, which are maintained through active glucose metabolism.

## Abstract 2

Redox homeostasis in the β cell ER is supported by a metabolic redox relay to maintain efficient insulin granule production.

## Introduction

The pancreatic islet β cell has evolved to convert physiological changes in ambient blood glucose to metabolic signals that control both insulin biosynthesis and insulin release (1–3). This essential function allows β cells to precisely manage nutrient uptake and storage in peripheral tissues via regulated insulin secretion. In response to rising blood glucose levels, glycolytic and TCA cycle activity increases in the β cell, which elicits an action potential via closure of the ATP-sensitive potassium channel, KIR6.1, that leads to Ca^2+^ influx and fusion of plasma membrane–docked insulin granules (4–7). Additional metabolites generated by glucose oxidation, including NADPH and glutathione, further amplify the actions of membrane depolarization (3, 8–10) to enhance insulin granule fusion via modifications to the exocytic machinery (10–13). Because β cells preferentially secrete newly formed insulin granules (14), β cells commit substantial resources to insulin production (1). Approximately 30%–50% of the total protein produced by the β cell is the insulin precursor, proinsulin, whose synthesis can be upregulated by more than 10-fold in response to glucose stimulation (15, 16). While the precise metabolic coupling mechanism is not known, sequences within the 5′-untranslated region of the preproinsulin mRNA confer glucose-stimulated translation (17). Taken together, these observations highlight multiple regulatory nodes within the β cell that utilize metabolism-derived signals to promote insulin secretion and insulin production.

In healthy β cells, approximately 20% of nascent proinsulin is misfolded and degraded, which is largely due to the difficulty in successfully forming the 3 essential disulfide linkages necessary for the insulin structure (18–20). Each disulfide linkage in proinsulin is derived from nonsequential cysteine residues in the primary amino acid sequence. This configuration presents a challenge in the oxidizing ER lumen because sequential cysteines readily form disulfide bonds. To correct non-native proinsulin confirmations, the cystine disulfides rely on isomerization via reduction and reoxidation to successfully achieve native proinsulin folding (18, 21, 22). Loss-of-function mutations in the ER oxidoreductases, *Ero1β*, *Ero1lβ*, and *Pdia1*, impair insulin production and increase ER stress susceptibility (19, 23, 24), whereas increased expression of *Ero1*α improves proinsulin folding and ER export (25). As an added challenge to proinsulin maturation, proinsulin dimerization in the ER requires both copies to be correctly folded prior to ER export (26, 27). Heterozygous mutations that perturb proinsulin folding act as dominant-negative alleles, leading to insulin insufficiency, β cell loss, and autosomal-dominant diabetes referred to as mutant *INS* gene–induced diabetes of youth (28, 29). In type 2 diabetes (T2D), increased levels of non-native proinsulin confirmations have been identified, which can catalyze the formation of high–molecular weight, disulfide-linked proinsulin aggregates (20, 30, 31). Whether the accumulation of proinsulin aggregates in T2D is pathological or nonreversible remains unknown, but substoichiometric levels of proinsulin folding mutants, such as C(A7)Y, are sufficient to negatively affect ER function, leading to β cell dysfunction and diabetes onset (32). Collectively, these observations highlight the need to better understand the regulation of β cell ER redox control, which may provide critical insight into β cell defects that contribute to the pathogenesis of diabetes.

We recently identified a trafficking delay in the ER export of proinsulin that leads to inadequate insulin granule production in rodent diabetes models (33). We showed that delayed proinsulin export results from hyperoxidation of the ER lumen and that restoration of ER redox homeostasis rescued proinsulin export and insulin granule formation. While this previous study provided a mechanism for how insulin deficiency develops in diabetes, the underlying mechanisms leading to the ER redox imbalance were not clear. In the present study, we demonstrate that impaired mitochondrial bioenergetics decreases the generation of reductive redox donors necessary to buffer ER redox homeostasis in pancreatic β cells. Our data correlate decreased NADPH flux with ER hyperoxidation and proinsulin trafficking delays in multiple β cell models of impaired mitochondrial function. We further show that limiting NADPH flux delays ER proinsulin export, which can be reversed using a cell-permeable reducing agent. In addition, suppression of thioredoxin reductase-1 (*Txnrd1*) impairs ER proinsulin export, whereas suppression of thioredoxin-interacting protein (*Txnip*), a negative regulator of thioredoxin, restores ER redox, proinsulin trafficking, and insulin secretion in models of β cell dysfunction. Taken together, our data suggest that β cell ER redox homeostasis is supported via a metabolic redox relay to maintain efficient insulin granule production.

## Results

### β Cell ER hyperoxidation accompanies impaired metabolic function.

**Figure 1:**
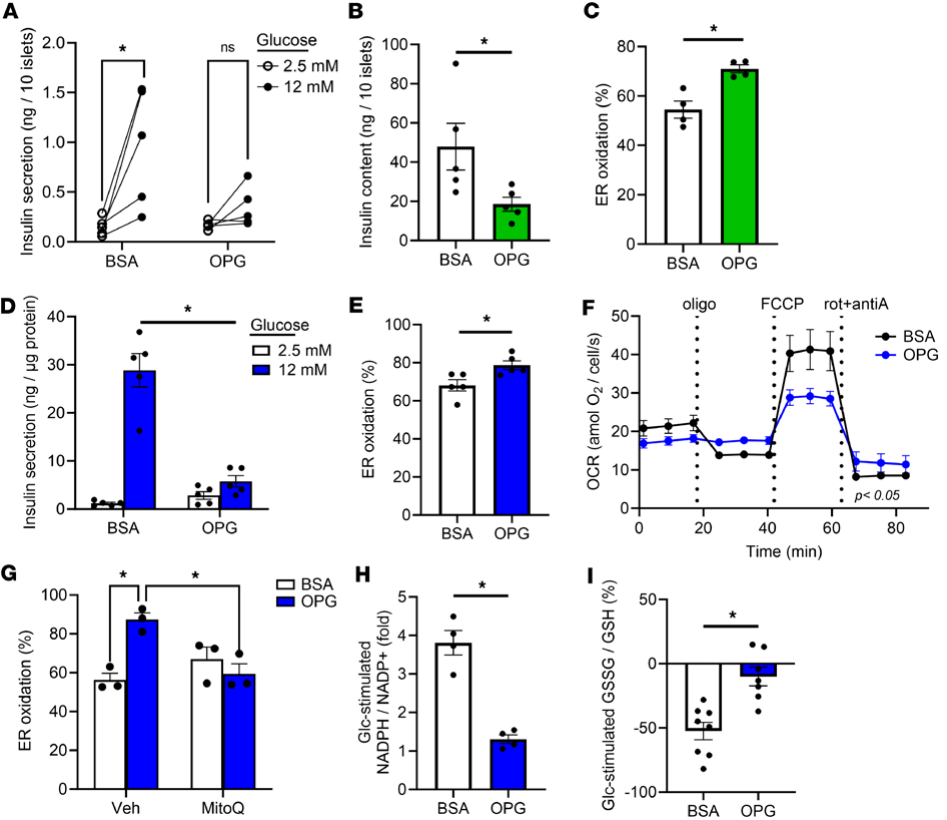
β Cell dysfunction and ER hyperoxidation coincide with impaired mitochondrial function and diminished cellular redox. Human islets (**A**–**C**) or INS-1 832/3 cells (**D**–**I**) were cultured for 3 days in control media supplemented with BSA (5.5 mM or 11 mM Glc, respectively) or media supplemented with oleate/palmitate (2:1, 1 mM) and elevated glucose (20 mM), labeled OPG, as indicated. (**A** and **D**) Insulin secretion was measured by static incubation in media containing 2.5 mM Glc followed by 12 mM Glc for 1 hour each (*n* = 5 human islet donors; *n* = 5 INS-1 experiments). (**B**) Insulin content was measured from human islet cell lysates (*n* = 5). (**C** and **E**) ER redox was determined from ratiometric imaging of cells expressing ERroGFP (AdRIP) (*n* = 4 or *n* = 5, respectively). (**F**) Oxygen consumption rate was measured following sequential addition of oligomycin A (2.5 μM), FCCP (1.25 μM), and rotenone plus antimycin A (2 μM each) as indicated (*n* = 3). (**G**) Cells were cocultured with MitoQ (0.5 μM) as indicated. ER redox was determined from ratiometric imaging of cells expressing ERroGFP (AdRIP) (*n* = 3). NADPH/NADP^+^ (**H**) or GSSG/GSH (**I**) were measured by sequential incubation in 2 mM Glc followed by 20 mM Glc for 12 minutes each via ratiometric imaging of iNAP (*n* = 4) or Grx1-roGFP2 (*n* = 7–8) (AdRIP), respectively. Responses were normalized to 2 mM Glc. (**A**–**I**) Data represent the mean ± SEM. *P < 0.05 by 2-way ANOVA with Tukey’s posttest analysis (**A** and **G**), 2-way ANOVA with Holm-Šídák posttest analysis (**D**), or 2-tailed Student’s *t* test (**B**, **C**, **E**, **H**, and **I**).

We previously demonstrated that hyperoxidation of the ER lumen impairs ER proinsulin export, leading to decreased insulin granule formation in rodent models of hyperglycemia and β cell dysfunction (33). In the current study, we tested if ER hyperoxidation also occurred in nondiabetic donor–derived human β cells cultured for 72 hours with elevated glucose (20 mM) and fatty acids termed OPG (1 mM oleate and palmitate, 2:1). Control islets were cultured with BSA carrier alone and 5.5 mM glucose. Note that the addition of oleate allows for prolonged exposure to fatty acid, which exacerbates β cell dysfunction but not β cell death (33–36). Culture in OPG perturbed glucose-stimulated insulin secretion (GSIS) and significantly decreased total insulin content in human islets (Figure 1, A and B). Ratiometric imaging of an ER-targeted redox-sensitive GFP reporter, ERroGFP, under control of the rat insulin promoter (RIP), revealed a significant shift to a more highly oxidized ER lumen in OPG-cultured human β cells (Figure 1C). Similarly, INS-1 832/3 cells cultured for 72 hours in OPG compared with BSA control cells had decreased GSIS (Figure 1D) and insulin content (Supplemental Figure 1A; supplemental material available online with this article; https://doi.org/10.1172/jci.insight.178725DS1). While proinsulin content was also decreased (Supplemental Figure 1B), the ratio of proinsulin to insulin was substantially increased (Supplemental Figure 1C). Furthermore, we observed ER hyperoxidation in OPG-cultured INS-1 cells (Figure 1E) as reported previously (33), whereas inhibition of the proteasome via MG132 was not sufficient to elicit ER hyperoxidation (Supplemental Figure 1, D–F). Importantly, ER hyperoxidation was fully reversible if OPG-cultured cells were recultured in control media (Supplemental Figure 1G).

Mitochondrial function and cellular redox shuttles can support ER redox in some cell types (37, 38). Although this connection has yet to be established in β cells, we speculated that perturbations in metabolic functions may contribute to the loss of ER redox control in β cells. To explore this hypothesis, we first used OPG-cultured INS-1 832/3 cells and observed a stark decrease in maximal respiratory capacity (carbonyl cyanide-p-trifluoromethoxyphenylhydrazone [FCCP] response) (Figure 1F). We then found that preservation of mitochondrial function using the mitochondria-targeted antioxidant MitoQ (also known as CoQ10), was sufficient to maintain ER redox in OPG-cultured INS-1 cells (Figure 1G). Next, we examined glucose-sensitive NADPH and glutathione redox cycles. Using the recently developed NADPH sensor, iNAP (39), we detected a strong glucose-dependent flux in NADPH cycling in BSA control cells that was severely attenuated in OPG-cultured cells (Figure 1H and Supplemental Figure 1H). The NADPH-insensitive iNAPc control sensor was glucose unresponsive, indicating that the signals detected by iNAP were not simply due to changes in cellular pH (Supplemental Figure 1I) (39). To assess NADPH availability, we examined glutathione redox cycling using the ratiometric sensor, Grx1-roGFP (40). The glucose-dependent shift of glutathione from the oxidized (GSSG) to the reduced (GSH) state present in BSA control cells was lost in OPG-cultured cells (Figure 1I and Supplemental Figure 1J). Collectively, these data are consistent with past reports of mitochondrial dysfunction and impaired NADPH redox cycles in T2D β cells and models of β cell dysfunction (10, 41) and correlate metabolic defects with ER hyperoxidation.

### Impaired mitochondrial function disrupts ER redox homeostasis and proinsulin trafficking.

**Figure 2:**
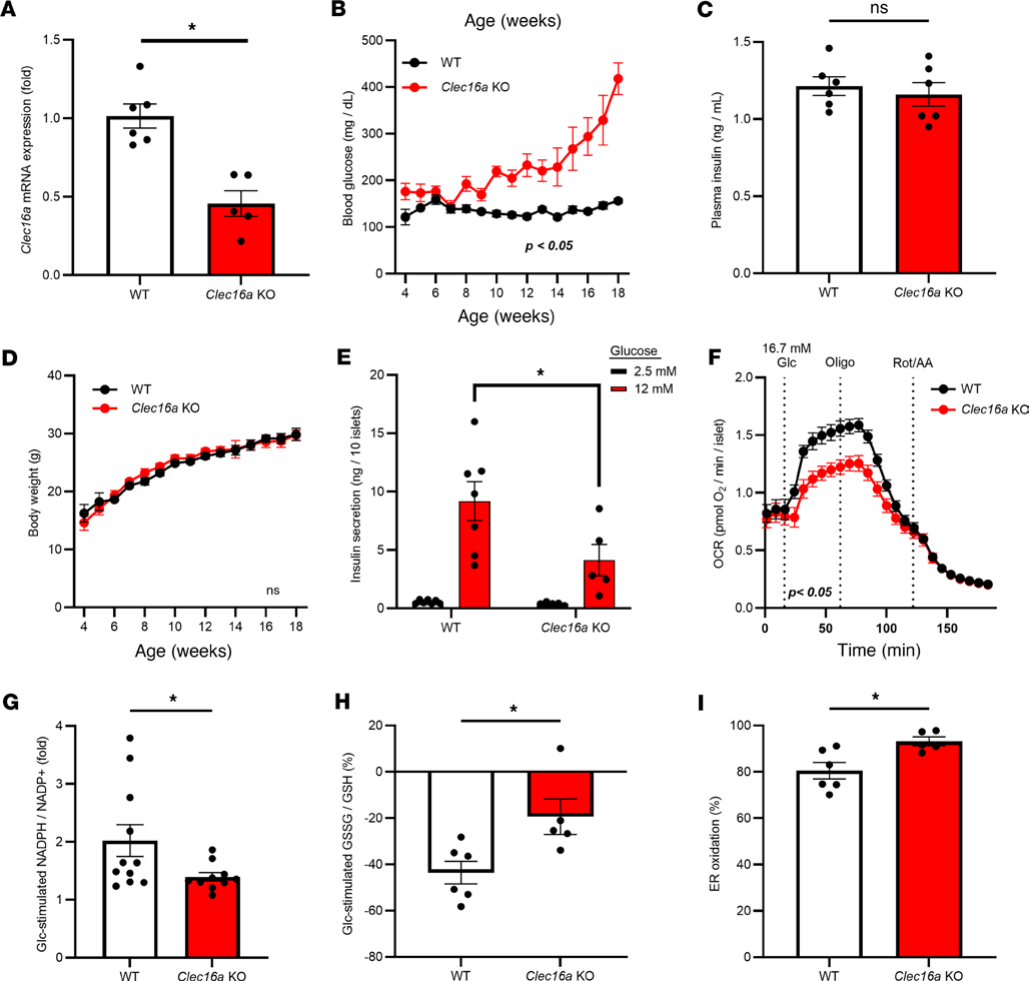
Mitochondrial dysfunction blunts cellular redox cycles with ER hyperoxidation. *Clec16a^fl/fl^* (WT) and *Clec16a^fl/fl^ Ins1-Cre* (*Clec16a*-KO) mice were used as follows. (**A**) mRNA expression of *Clec16a* was determined by RT-qPCR from isolated islets from 18-week-old female mice (*n* = 5–6). (**B**) Ad lib–fed blood glucose was monitored weekly in male mice beginning at 4 weeks of age (*n* = 10). (**C**) Plasma insulin was measured from ad lib–fed 14-week-old male mice (*n* = 6). (**D**) Body weight was monitored in male mice beginning at 4 weeks of age (*n* = 10). (**E**) Insulin secretion was measured in islets from 14-week-old male mice by static incubation at 2.5 mM Glc followed by 12 mM Glc for 1 hour each (*n* = 5–7). (**F**) Oxygen consumption rate was measured in islets from 10-week-old male mice (*n* = 5) via Seahorse respirometry at basal glucose (2 mM), stimulatory glucose (16.7 mM), oligomycin (5 μM), and rotenone plus antimycin A (5 μM each) as indicated. NADPH/NADP^+^ (**G**, *n* = 10–11) and GSSG/GSH (**H**, *n* = 5–6) were measured in islets from 14- to 20-week-old male and female mice by sequential incubation in 2 mM Glc followed by 20 mM Glc for 12 minutes each via ratiometric imaging of iNAP or Grx1-roGFP2 (AdRIP), respectively. Responses were normalized to 2 mM Glc. (**I**) ER redox was measured in islets from 8-week-old male mice (*n* = 5–6) via ratiometric imaging of cells expressing ERroGFP (AdRIP). (**A**–**I**) Data represent the mean ± SEM. *P < 0.05 by 2-tailed Student’s *t* test (**A**, **C**, and **G**–**I**), 2-way ANOVA with mixed-model posttest analysis (**B** and **D**), 2-way ANOVA with Holm-Šídák posttest analysis (**E**), or 2-way ANOVA with repeated measures analysis (**F**).

To test if decreased mitochondrial bioenergetics are sufficient to elicit ER hyperoxidation, we used a β cell–specific knockout (KO) of *Clec16a*, which regulates mitophagy, a quality control mechanism to remove damaged mitochondria (42–44). Mitochondrial damage in *Clec16a^fl/fl^ Ins1^tm1.1(cre)Thor^* (β cell *Clec16a*-KO) mice triggers a gradual decline in β cell function and diabetes onset within 4 months without dietary intervention or insulin resistance. Using isolated islets from β cell *Clec16a*-KO mice compared with wild-type littermate controls (*Clec16a^fl/fl^*), we verified loss of *Clec16a* expression by reverse transcription quantitative PCR (RT-qPCR) (Figure 2A). The remaining expression is likely due to *Clec16a* in non–β cells of the islet or incomplete penetrance of the *Ins1-Cre* transgene. Within 8 weeks of age, ad lib–fed hyperglycemia was evident in β cell *Clec16a*-KO mice that steadily worsened with age (Figure 2B and Supplemental Figure 2A), whereas plasma insulin failed to compensate (Figure 2C and Supplemental Figure 2B). Body weight did not differ between genotypes (Figure 2D and Supplemental Figure 2C).

We next explored metabolic functions in β cell *Clec16a*-KO mice at 10–18 weeks of age to avoid potential confounding issues related to loss of β cell identity (42). Using isolated islets, we showed that β cell *Clec16a* KOs had impaired GSIS (Figure 2E and Supplemental Figure 2D), which was present across multiple glucose concentrations (Supplemental Figure 2E). Importantly, the *Ins1-Cre* alone does not affect glucose tolerance, insulin content, or β cell function (45). The secretory impairment in β cell *Clec16a*-KO islets coincided with a substantial decrease in mitochondrial oxygen consumption (Figure 2F), as previously shown (44). Using the adenovirally delivered iNAP ratiometric sensor, we observed a substantial loss in glucose-stimulated NADPH flux (Figure 2G and Supplemental Figure 2F) that coincided with impaired glutathione redox cycling (Figure 2H and Supplemental Figure 2G). Furthermore, these metabolic defects were accompanied by significant ER hyperoxidation (Figure 2I) yet no evidence of overt ER stress or changes in oxidoreductase gene expression (Supplemental Figure 2H).

**Figure 3:**
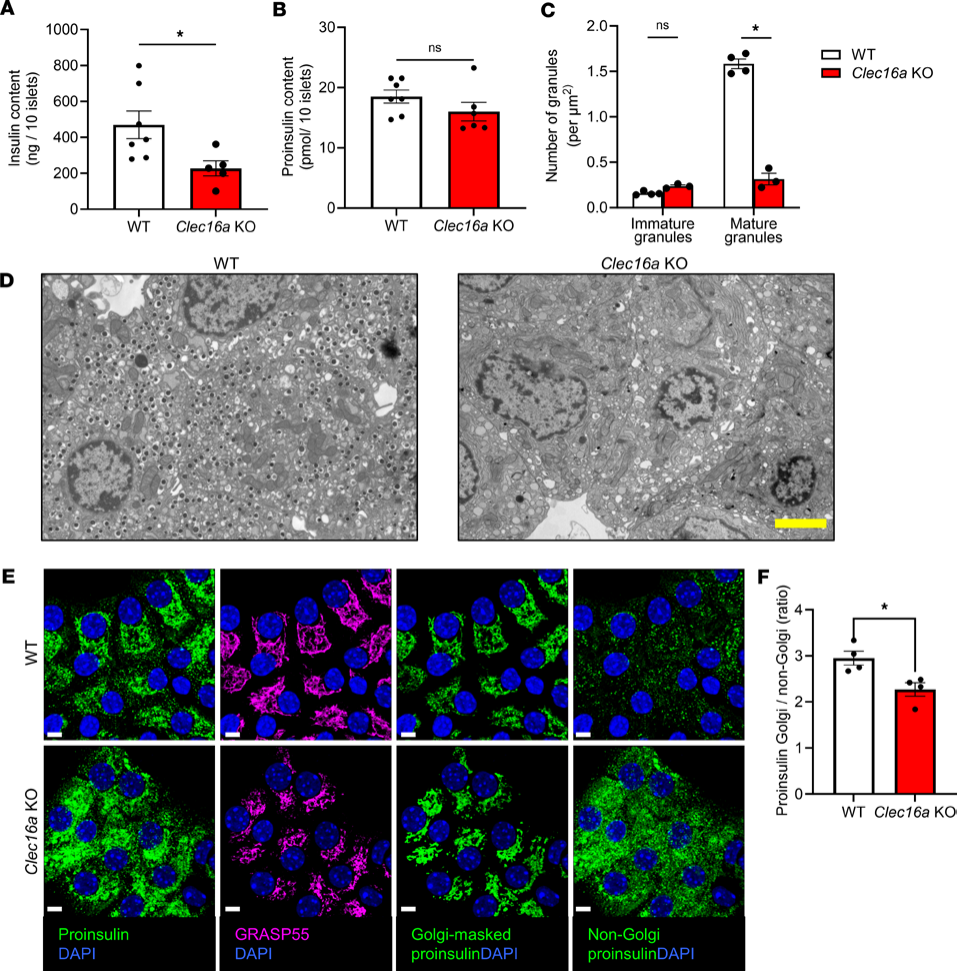
Mitochondrial dysfunction leads to decreased insulin content. *Clec16a^fl/fl^* (WT) and *Clec16a^fl/fl^ Ins1-Cre* (*Clec16a*-KO) mice were used as follows. Insulin (**A**) and proinsulin (**B**) content was determined from islet cell lysates from 8-week-old male mice (*n* = 5–7). (**C** and **D**) Isolated islets from 13-week-old male mice were imaged by TEM. (**C**) The numbers of immature and mature granules per cell area were quantified (*n* = 3–4). (**D**) Representative electron micrographs are shown. (**E** and **F**) Islets from 18-week-old mice were fixed, immunostained for proinsulin (green) and GRASP55 (Golgi, magenta), and counterstained with DAPI (blue). Using a region of interest mask derived from the GRASP55 staining (magenta), proinsulin (green) contained within the Golgi (Golgi-masked proinsulin) and outside of the Golgi (non-Golgi proinsulin) was determined. (**E**) Representative images are shown. (**F**) The ratio of proinsulin coincident with the Golgi versus non-Golgi region was quantified. (**A**, **B**, **C**, and **F**) Data represent the mean ± SEM. *P < 0.05 by 2-tailed Student’s *t* test (**A**, **B**, and **F**) or 2-way ANOVA with Holm-Šídák posttest analysis (**C**). Scale bar = 3 μm.

We previously demonstrated that ER hyperoxidation impairs proinsulin trafficking, leading to a loss of insulin secretory granules in models of hyperglycemia and β cell dysfunction (33). Based on this, we tested if ER hyperoxidation in β cell *Clec16a*-KO islets also coincided with alterations to proinsulin trafficking. We showed that insulin content was strongly decreased in *Clec16a*-KO islets (Figure 3A and Supplemental Figure 3A), yet proinsulin content was not different between genotypes (Figure 3B and Supplemental Figure 3B). Examination of cellular ultrastructure by transmission electron microscopy (TEM) verified a marked decrease in mature insulin granules in *Clec16a*-KO β cells, with no difference in immature granule number (Figure 3, C and D). Note that the loss of insulin granules in *Clec16a*-KO mice was previously reported using *Pdx1-Cre*, though total pancreatic insulin content was increased (44).

Using immunostaining and confocal imaging, we next examined the localization of proinsulin in *Clec16a*-KO β cells. For quantitative analysis, the fluorescence intensity of proinsulin within the Golgi was measured using a region of interest mask defined by GRASP55 staining (Golgi, magenta) and compared with the remaining non-Golgi cellular staining (Figure 3, E and F). In WT β cells, proinsulin was primarily localized within the Golgi, whereas a significant portion of proinsulin was also outside the Golgi region in *Clec16a*-KO β cells. Because no difference in immature granules or proinsulin content was observed (Figure 3, B and C), we speculated that the non-Golgi proinsulin resides in the ER as previously reported for poorly trafficked and misfolded proinsulin in diabetes models (27, 28, 33, 46).

**Figure 4:**
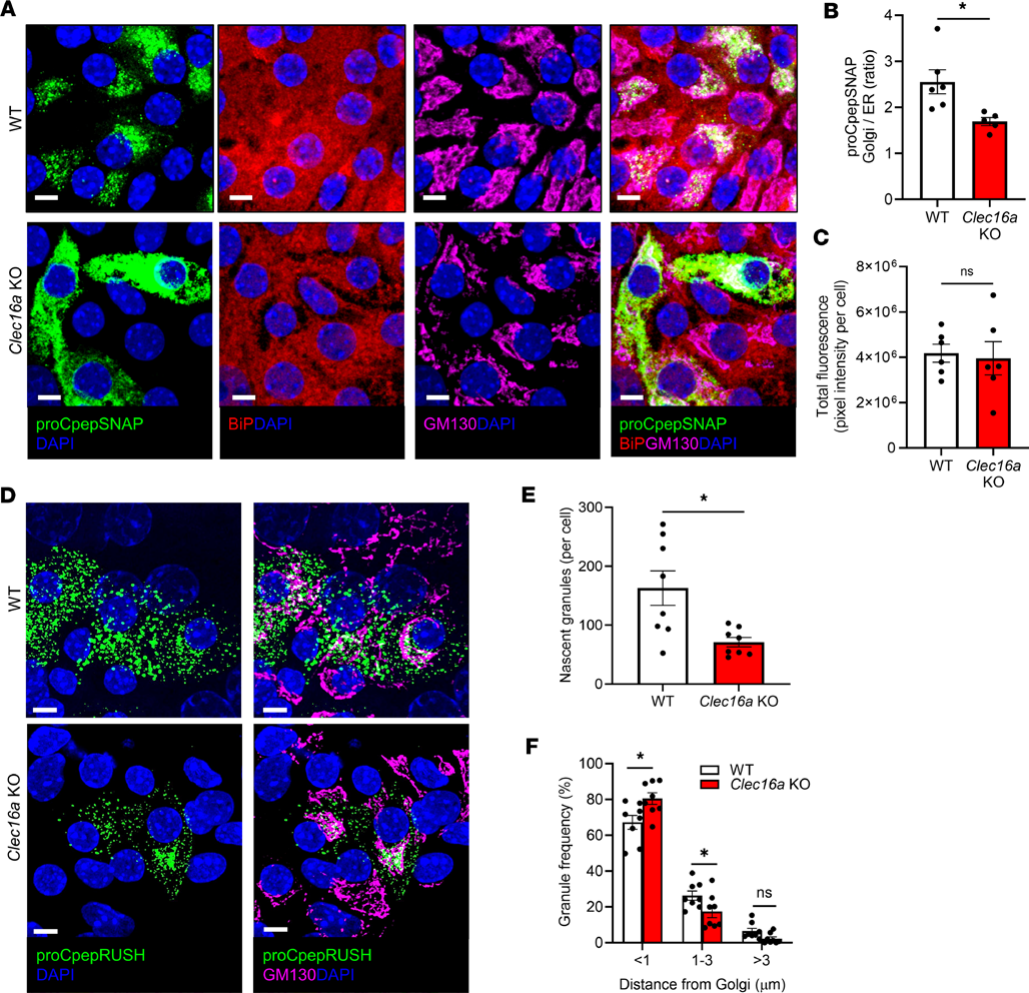
Mitochondrial dysfunction impairs ER-Golgi proinsulin trafficking. *Clec16a^fl/fl^* (WT) and *Clec16a^fl/fl^ Ins1-Cre* (*Clec16a*-KO) mice were used as follows. (**A**–**C**) Islets from 13- to 18-week-old male mice were treated with AdRIP-proCpepSNAP. At 48 hours after infection, islets were pulse-labeled with SNAP-505 (green), chased for 10 minutes, immunostained for BiP (red) and GM130 (magenta), and counterstained with DAPI (blue). (**A**) Representative images are shown. (**B**) The ratio of proCpepSNAP fluorescence coincident with the Golgi (GM130) compared with ER (BiP) was quantified (*n* = 5–6). (**C**) Total fluorescence intensity of proCpepSNAP was quantified. (**D**–**F**) Islets from 8- to 10-week-old male and female mice expressing proCpepRUSH (AdRIP) were treated with biotin (200 μM) to initiate trafficking. At 3 hours after biotin addition, cells were fixed, immunostained for GM130 (Golgi, magenta), and counterstained with DAPI (blue). (**D**) Representative images are shown. (**E**) The number of insulin granules per cell was quantified (*n* = 8). (**F**) Insulin granule distance from the nearest point on the Golgi was quantified as a frequency per binned distance (*n* = 8). Data represent the mean ± SEM. *P < 0.05 by 2-tailed Student’s *t* test (**B**, **C**, and **E**) or 2-way ANOVA with Holm-Šídák posttest analysis (**F**). Scale bar = 5 μm.

To directly examine proinsulin trafficking in β cell *Clec16a* KOs, we used the in situ fluorescent pulse-chase proinsulin reporter system, proCpepSNAP. In this system, SNAPtag is inserted within the C-peptide region of human proinsulin and can be pulse-labeled by addition of a cell-permeable substrate. Previous studies have demonstrated the proCpepSNAP reporter mimics the trafficking, processing, and secretion of endogenous proinsulin/insulin (33, 47–49). Importantly, this reporter is delivered by recombinant adenovirus and driven by the RIP to ensure β cell–selective expression. Given the alteration to ER redox (Figure 2I) and proinsulin immunostaining (Figure 3, E and F), we focused on ER export by evaluating the transit of proinsulin from the ER to Golgi (33). Within 10 minutes of pulse-chase in WT control β cells, nascent proinsulin (proCpepSNAP) was coincident with the cis-Golgi marker, GM130 (Figure 4, A and B). In contrast, nascent proinsulin (proCpepSNAP) remained predominantly localized with the ER marker, BiP, in *Clec16a*-KO β cells. The specificity of the BiP antibody was verified by siRNA knockdown (Supplemental Figure 4). Quantification of proCpepSNAP localization using region of interest masks defined by GM130 and BiP staining verified a clear decrease in the ratio of Golgi to ER proCpepSNAP staining in *Clec16a*-KO β cells (Figure 4B and Supplemental Figure 5A). Note that total proCpepSNAP labeling did not differ between genotypes (Figure 4C). These data suggest that ER export of proinsulin is delayed in *Clec16a*-KO β cells.

To explore the impact of delayed ER export of proinsulin on insulin granule formation, we used the synchronized proinsulin trafficking system, proCpepRUSH (48). Note, this trafficking system is based on the reversible interaction of the ER-localized hook, streptavidin-KDEL, with proinsulin containing a streptavidin-binding peptide and GFP (proCpepRUSH), which allows for synchronous release of proinsulin from the ER via addition of biotin. Using proCpepRUSH, we observed substantially fewer insulin granules produced in β cell *Clec16a* KOs following a 3-hour chase (Figure 4, D and E), with a significant proportion of proCpepRUSH staining proximal to the Golgi (Figure 4F). No difference in proCpepRUSH expression between genotypes was detected (Supplemental Figure 5B). Taken together, these data demonstrate that loss of mitochondrial function is sufficient to elicit ER hyperoxidation and delay ER proinsulin export, leading to defects in insulin granule formation.

### Acute metabolic inhibition impairs ER redox and proinsulin ER exit.

**Figure 5:**
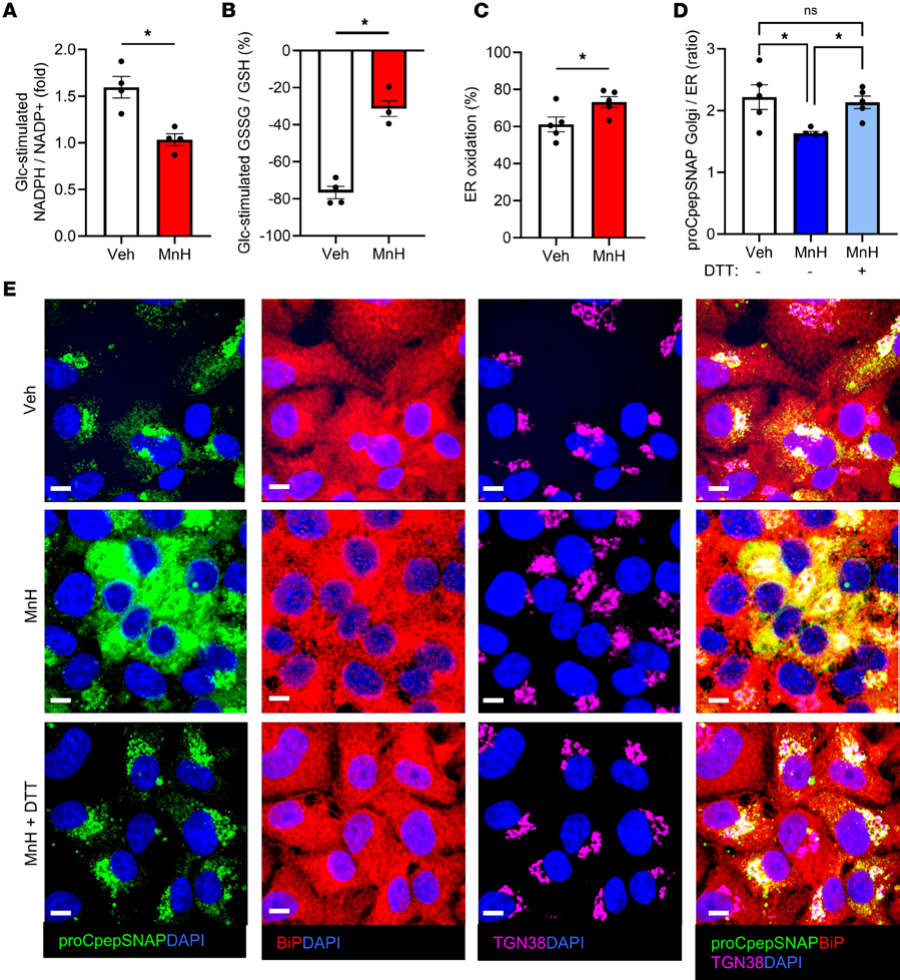
Pharmacological inhibition of metabolic activity triggers ER hyperoxidation and delayed ER-Golgi proinsulin transport. Male and female 12- to 16-week-old mouse islets (**A**–**C**) or INS-1 832/3 cells (**D** and **E**) were treated with vehicle (veh) or mannoheptulose (MnH; 2 mM or 1 mM, respectively) for 4 hours as indicated. NADPH/NADP^+^ (**A**, *n* = 4) and GSSG/GSH (**B**, *n* = 4) were measured by sequential incubation in 2 mM Glc followed by 20 mM Glc for 12 minutes each via ratiometric imaging of iNAP or Grx1-roGFP2 (AdRIP), respectively. Responses were normalized to 2 mM Glc. (**C**) ER redox was measured (*n* = 5) via ratiometric imaging of ERroGFP (AdRIP). (**D** and **E**) INS-1 832/3 cells stably expressing proCpepSNAP cells were treated with veh or MnH or cotreated with MnH plus DTT (0.5 mM; MnH + DTT) for 4 hours as indicated. Cells were pulse-labeled with SNAP-505 (green), chased for 10 minutes, immunostained for TGN38 (magenta) and BiP (red), and counterstained with DAPI (blue). (**D**) The ratio of proCpepSNAP fluorescence coincident with the Golgi (TGN38) versus ER (BiP) was quantified (*n* = 4). (**E**) Representative images are shown. (**A**–**D**) Data represent the mean ± SEM. *P < 0.05 by 2-tailed Student’s *t* test (**A**–**C**) or 1-way ANOVA with Tukey’s posttest analysis (**D**). Scale bar = 5 μm.

To further explore the relationship between metabolic activity, cellular redox, and ER redox, we examined the impact of acute (4 hours) metabolic suppression via the glucokinase inhibitor, mannoheptulose. As expected, mannoheptulose treatment decreased cellular respiration but was less severe than the complex I inhibitor, rotenone (Supplemental Figure 6A). In primary mouse β cells, mannoheptulose treatment diminished glucose-stimulated NADPH flux (Figure 5A and Supplemental Figure 6B), which was accompanied by decreased glutathione redox cycling (Figure 5B and Supplemental Figure 6C). Furthermore, acute mannoheptulose treatment led to hyperoxidation of the ER lumen in both mouse islet β cells (Figure 5C) and INS-1 832/3 cells (Supplemental Figure 7A). Next, we investigated ER-Golgi trafficking of proinsulin using proCpepSNAP. Following pulse-chase labeling, we observed that mannoheptulose resulted in a marked retention of nascent proinsulin (proCpepSNAP) with the ER marker, BiP, that was distinct from the predominant Golgi (TGN38) localization of nascent proinsulin in vehicle controls (Figure 5, D and E, and Supplemental Figure 7D), with no difference in proCpepSNAP intensity between conditions (Supplemental Figure 7B). Direct comparison of acute mannoheptulose treatment with prolonged (72 hours) culture in OPG revealed a similar proinsulin trafficking delay (Supplemental Figure 7C).

We previously demonstrated that cellular reducing equivalents can restore ER redox and rescue ER-Golgi proinsulin trafficking in models of diet-induced β cell dysfunction (33). In these experiments, DTT (0.5 mM) or vehicle control was added 4 hours prior to pulse-chase labeling, which does not elicit an ER stress response. Consistent with our previous report (33), DTT supplementation was able to rescue ER-Golgi proinsulin trafficking in mannoheptulose-treated mouse β cells (Figure 5, D and E and Supplemental Figure 7D). Collectively, our data suggest that metabolic activity supplies critical reducing donors to support ER redox control for efficient proinsulin trafficking.

### Diminished NADPH flux disrupts ER redox homeostasis and proinsulin trafficking.

**Figure 6:**
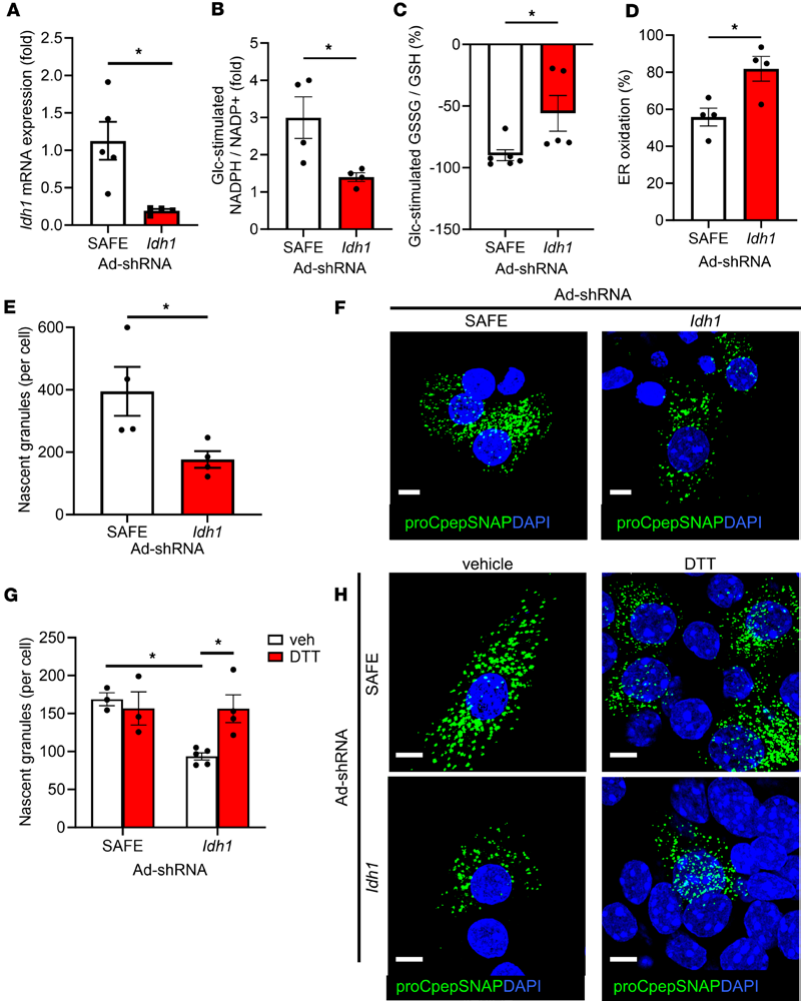
*Idh1* suppression elicits ER hyperoxidation and impaired insulin granule formation. Male and female 12- to 16-week-old mouse islets were treated with Ad-shSAFE or Ad-sh*Idh1* as indicated. (**A**) mRNA expression of *Idh1* was determined by RT-qPCR (*n* = 5). NADPH/NADP^+^ (**B**, *n* = 4) and GSSG/GSH (**C**, *n* = 5–6) were measured in mCherry^+^ islet cells (Ad-shRNA) by sequential incubation in 2 mM Glc followed by 20 mM Glc for 12 minutes each via ratiometric imaging of iNAP or Grx1-roGFP2 (AdRIP), respectively. Responses were normalized to 2 mM Glc. (**D**) ER redox was measured in mCherry^+^ islet cells (Ad-shRNA; *n* = 4) via ratiometric imaging of ERroGFP (AdRIP). (**E** and **F**) ProCpepSNAP-expressing islets (AdRIP) were pulse-labeled with SNAP-505 (green) and chased for 2 hours. Cells were fixed and counterstained with DAPI (blue). The total number of nascent (proCpepSNAP-labeled) granules per mCherry^+^ cell was quantified (**E**, *n* = 4) and representative images are shown (**F**). (**G** and **H**) ProCpepSNAP-expressing islets (AdRIP) were cultured with vehicle (control) or DTT (0.5 mM) for 4 hours prior to pulse-labeling with SNAP-505 (green) and chased for 2 hours. Cells were fixed and counterstained with DAPI (blue). The total number of nascent (proCpepSNAP-labeled) granules per mCherry^+^ cell was quantified (**G**, *n* = 3–5) and representative images are shown (**H**). (**A**–**E** and **G**) Data represent the mean ± SEM. *P < 0.05 by 2-tailed Student’s *t* test (**A**–**E**) or 2-way ANOVA with Holm-Šídák posttest analysis (**G**). Scale bar = 5 μm.

Based on our data using models of metabolic dysfunction, we speculated that metabolic regulation of NADPH flux functions in a redox donor shuttle to support β cell ER redox homeostasis. To test this, we used shRNA knockdown of cytosolic isocitrate dehydrogenase-1 (*Idh1*), which regulates glucose-stimulated NADPH flux in β cells (50, 51). Control islets were treated with virus expressing nontargeting shSAFE (Figure 6A). Importantly, *Idh1* loss does not impair mitochondrial function (51). The shRNA viral backbone coexpresses mCherry for identification of shRNA-expressing cells. Consistent with previous reports (10, 51), shRNA KD of *Idh1* strongly decreased glucose-stimulated NADPH cycling in primary mouse β cells (Figure 6B and Supplemental Figure 8A), which was accompanied by a decrease in glutathione redox cycling (Figure 6C and Supplemental Figure 8B). Furthermore, *Idh1* suppression resulted in hyperoxidation of the ER lumen (Figure 6D).

We next examined whether *Idh1* KD impaired proinsulin trafficking in primary mouse β cells via fluorescent pulse-chase labeling of the proCpepSNAP reporter. In these studies, we examined proinsulin delivery into nascent insulin granules following a 2-hour chase as a measure of successful proinsulin trafficking. In *Idh1*-KD β cells, we observed a significant (>50%) decrease in the production of nascent insulin granules, identified as proCpepSNAP-labeled puncta, compared with Ad-shSAFE–treated β cells (Figure 6, E and F), with no difference in proCpepSNAP labeling between groups (Supplemental Figure 8C). To explore if the impairment in proinsulin trafficking was linked to ER hyperoxidation, we tested whether DTT addition could restore insulin granule formation in *Idh1*-KD β cells. Consistent with our previous data (33), addition of DTT compared with vehicle control did not affect insulin granule formation in Ad-shSAFE–treated cells (Figure 6, G and H) or proCpepSNAP fluorescence (Supplemental Figure 8D). In contrast, treatment of *Idh1* shRNA–KD β cells with DTT rescued insulin granule formation, restoring the levels to the Ad-shSAFE control. Taken together, our data indicate that ER redox poise and proinsulin trafficking can be regulated by NADPH flux and reducing donor availability.

### Thioredoxin contributes to β cell ER redox control.

**Figure 7:**
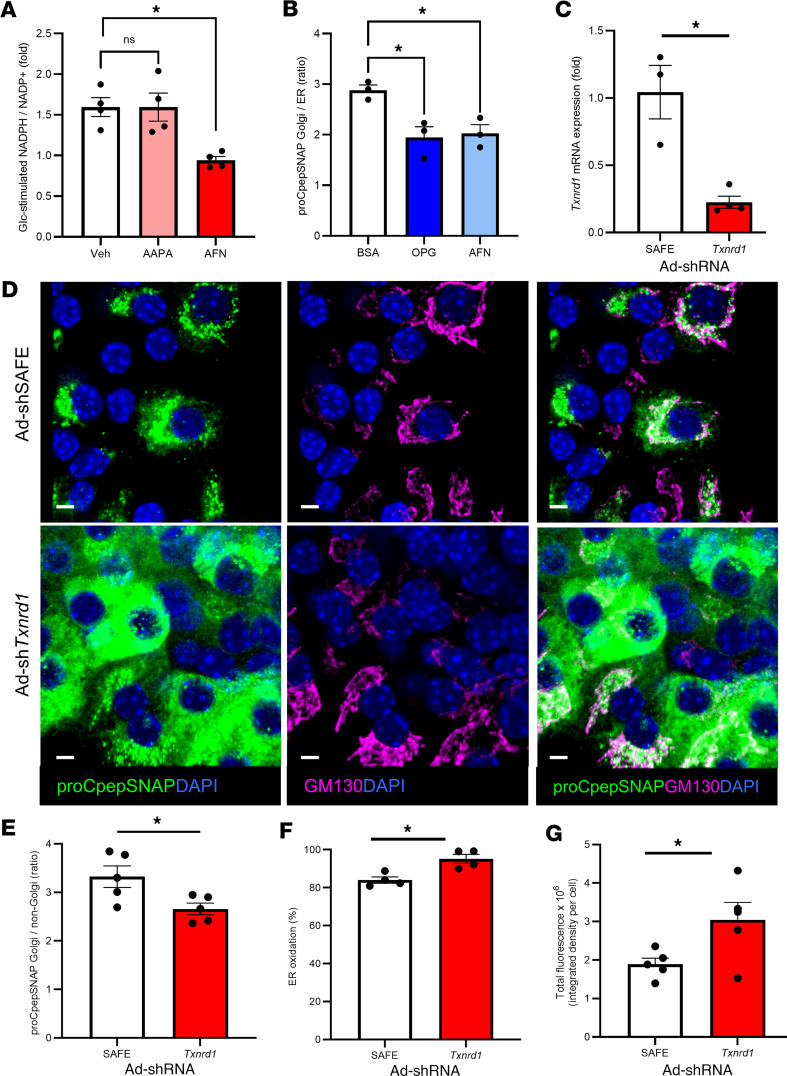
Inhibition of GSR1 or TXNRD1 impairs ER redox homeostasis and ER-Golgi transport of proinsulin. (**A** and **B**) Male and female 12- to 16-week-old mouse islets or INS-1 832/3 cells were treated with vehicle (veh), 2-AAPA (25 μM), or auranofin (AFN; 10 μM) for 4 hours before imaging. (**A**) NADPH/NADP^+^ were measured in islets (*n* = 4) by sequential incubation in 2 mM Glc followed by 20 mM Glc for 12 minutes each via ratiometric imaging of iNAP (AdRIP). Responses were normalized to 2 mM Glc. (**B**) INS-1 832/3 cells stably expressing proCpepSNAP were pulse-labeled with SNAP-505, chased for 10 minutes, immunostained for BiP and TGN38, and counterstained with DAPI. The ratio of proCpepSNAP fluorescence coincident with the Golgi (TGN38) versus the ER (BiP) was quantified (*n* = 3). (**C**–**G**) Male and female 12- to 16-week-old mouse islets were treated with Ad-shSAFE or Ad-sh*Txnrd1* as indicated and analyzed 96 hours after infection. (**C**) *Txnrd1* mRNA expression was quantified by RT-qPCR (*n* = 3–4). (**D**, **E**, and **G**) Male and female 12- to 16-week-old mouse islets expressing proCpepSNAP (AdRIP) were pulse-labeled with SNAP-505 (green) and chased for 10 minutes. Cells were fixed, immunostained for GM130 (magenta), and counterstained for DAPI (blue). Representative images are shown (**D**) and the ratio of proCpepSNAP coincident with the Golgi (GM130) versus non-Golgi region in mCherry^+^ cells (*n* = 5) was quantified (**E**) and total fluorescence intensity calculated (**G**). (**F**) ER redox was measured in mCherry^+^ islet cells (Ad-shRNA; *n* = 4) via ratiometric imaging of ERroGFP (AdRIP). (**A**–**C** and **E**–**G**) Data represent the mean ± SEM. *P < 0.05 by 1-way ANOVA with Dunnett’s posttest analysis (**A**), 1-way ANOVA with Tukey’s posttest analysis (**B**), or 2-tailed Student’s *t* test (**C** and **E**–**G**). Scale bar = 5 μm.

Our data correlate loss of glutathione redox cycling with ER hyperoxidation in β cells; however, both major redox donors, glutathione and thioredoxin, can contribute to ER redox control and are preferentially utilized in some cell types (37, 38, 52). To explore these redox cycles, we focused on the cytosolic NADPH-dependent enzymes, glutathione reductase-1 (GSR1) and TXNRD1, which regulate glutathione and thioredoxin redox cycles, respectively. We first used pharmacological inhibition of GSR1 or TXNRD1 and determined the impact on NADPH cycling. While inhibition of TXNRD1 using auranofin eliminated glucose-stimulated NADPH flux in mouse β cells (Figure 7A and Supplemental Figure 9A) similar to metabolic inhibition (Figure 1H, Figure 2G, and Figure 5A), the GSR1 inhibitor, 2-AAPA, had no effect on NADPH cycling. Based on these data, we next examined the impact of TXNRD1 inhibition on ER proinsulin export using proCpepSNAP pulse-chase labeling. Acute TXNRD1 inhibition by auranofin delayed ER-Golgi proinsulin transport similar to the impact of chronic OPG culture of INS-1 832/3 cells (Figure 7B and Supplemental Figure 9B). To further test this, we used adenovirus-delivered shRNA to suppress *Txnrd1* in primary mouse β cells (Figure 7C). KD of *Txnrd1* resulted in accumulation of proCpepSNAP localized outside of the Golgi staining region (Figure 7, D and E, and Supplemental Figure 10) and was accompanied by ER hyperoxidation (Figure 7F). Note, increased labeling of proCpepSNAP was observed in *Txnrd1*-KD cells (Figure 7G); however, the relative localization was strongly biased to the non-Golgi region (Figure 7, D and E).

**Figure 8:**
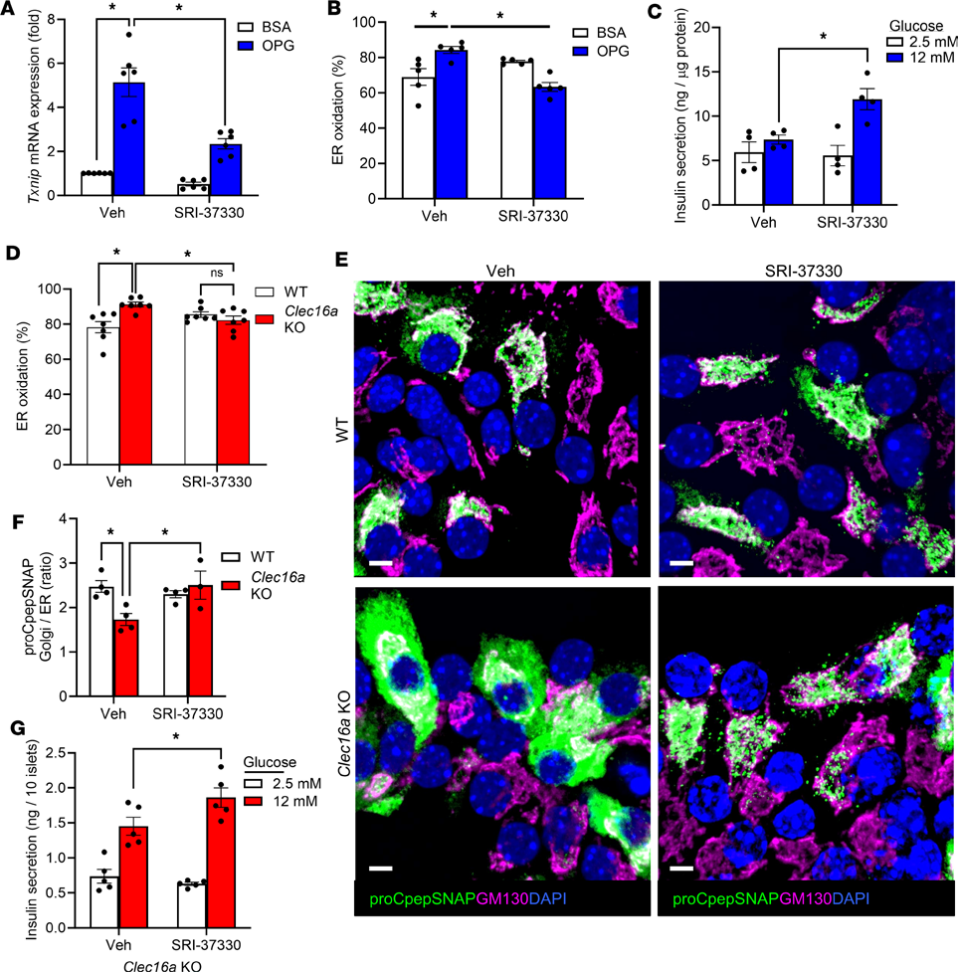
*Txnip* suppression restores ER redox homeostasis, proinsulin trafficking, and β cell function. (**A**–**C**) INS-1 832/3 cells were cultured for 72 hours in BSA or OPG with either vehicle (veh) control or SRI-37330 (1 μM) added in the last 24 hours. (**A**) *Txnip* mRNA expression was quantified by RT-qPCR (*n* = 6). (**B**) ER redox was measured via ratiometric imaging of ERroGFP (AdRIP) (*n* = 5). (**C**) Insulin secretion was measured by static incubation in media containing 2.5 mM Glc followed by 12 mM Glc for 1 hour each (*n* = 4). (**D**–**G**) Islets from male *Clec16a^fl/fl^* (WT) and *Clec16a^fl/fl^* *Ins1-Cre* (*Clec16a*-KO) mice (12–16 weeks old) were treated with SRI-37330 (1 μM) for 24 hours. (**D**) ER redox was measured via ratiometric imaging of ERroGFP (AdRIP) (*n* = 7). (**E** and **F**) Islets expressing proCpepSNAP (AdRIP) were pulse-labeled with SNAP-505 (green) and chased for 10 minutes. Cells were fixed, immunostained for GM130 (magenta) and BiP, and counterstained with DAPI (blue). Representative images are shown (**E**) and the ratio of proCpepSNAP (green) fluorescence coincident with the Golgi (GM130) versus ER (BiP) was quantified (**F**). (**G**) Insulin secretion was measured by static incubation in media containing 2.5 mM Glc followed by 12 mM Glc for 1 hour each (*n* = 5). (**A**–**D**, **F**, and **G**) Data represent the mean ± SEM. *P < 0.05 by 2-way ANOVA with Tukey’s posttest analysis (**A**, **B**, **D**, and **F**) or 2-way ANOVA with Holm-Šídák posttest analysis (**C** and **G**). Scale bar = 5 μm.

To further explore the contribution of thioredoxin to the regulation of β cell ER redox homeostasis, we examined TXNIP, which directly binds and inhibits thioredoxin, leading to oxidative stress, inflammasome activation, and β cell apoptosis (53–55). TXNIP is highly upregulated in response to hyperglycemia in models of β cell dysfunction (53), including chronic culture in OPG (Figure 8A) and β cell *Clec16a*-KO mice (Supplemental Figure 11A). Whether TXNIP affects ER redox or proinsulin trafficking is not known. To examine this possibility, we used the recently developed inhibitor of *Txnip* expression, SRI-37330 (56), and showed that overnight *Txnip* suppression (Figure 8A) could restore ER redox homeostasis in OPG-cultured INS-1 832/3 cells (Figure 8B) and improve GSIS (Figure 8C). Note that these beneficial effects did not occur through improvements to upstream NADPH cycling (Supplemental Figure 11, B and C). Furthermore, SRI-37330 treatment also restored ER redox in *Clec16a*-KO β cells (Figure 8D). This was accompanied by rescue of ER-Golgi trafficking of proinsulin (Figure 8, E and F, and Supplemental Figure 12) and an improvement in GSIS (Figure 8G). No alterations to GSIS or insulin content were observed in SRI-37330–treated islets from WT (*Clec16a^fl/fl^*) mice (Supplemental Figure 11, D and E), nor were there changes in proCpepSNAP intensity between groups (Supplemental Figure 11F). Collectively, these data demonstrate that ER redox capacity in the β cell is regulated by the metabolic supply of reductive redox donors, such as thioredoxin, and highlight the potential utility of targeting antioxidant pathways in the restoration of proinsulin trafficking and β cell secretory function.

## Discussion

In β cells, NADPH-dependent cellular redox cycles, including glutathione and thioredoxin, are strongly coupled with glucose metabolism and serve as a cytosolic currency of TCA cycle activity to couple β cell functions with rates of glucose metabolism (3, 57). For example, NADPH-dependent reduction of glutathione functions in a redox shuttle to activate sentrin/SUMO-specific protease-1 (SENP1), which amplifies GSIS via modifications to the exocytic machinery (10–13). In this report, we show that glucose metabolism and cellular redox cycles also contribute to ER redox homeostasis and proinsulin trafficking. This mechanism allows for parallel regulation of the insulin exocytic and biosynthetic pathways as well as providing insight into the metabolic dysregulation of insulin release and production in T2D. Our data correlate alterations to NADPH flux with ER hyperoxidation and proinsulin trafficking delays in multiple models of impaired mitochondrial function. We show that limiting NADPH and thioredoxin redox elicits ER hyperoxidation and impairs proinsulin trafficking. In contrast, restoration of ER redox homeostasis via supplementation with reducing agent or *Txnip* suppression promotes proinsulin trafficking in multiple models. Based on these data, we propose that β cell ER redox capacity is maintained via glucose-dependent activation of NADPH and thioredoxin redox cycles to ensure ER folding capacity meets the biosynthetic demands of glucose-stimulated insulin production.

Our work builds upon an emerging concept that oxidative protein folding in the ER can be regulated via nutrient metabolism through the activation of redox cycles (37, 38, 52, 58). Redox donors, such as thioredoxin, can assist protein disulfide isomerases in forming native disulfide bonds and reducing non-native bonds (59), referred to as redox buffering. This activity ensures successful proinsulin folding via iterative rounds of disulfide bond isomerization. The precise mechanism for how thioredoxin is used as a redox buffer for the ER remains to be determined but is suggested to utilize an ER membrane carrier as a redox relay (60). While direct measures of thioredoxin redox cycles in live cells remains challenging, our proposed mechanism is supported by pharmacological inhibition of TXNRD1 and suppression of *Txnip* and *Txnrd1*. In further support of this mechanism, *Txnrd1* β cell–KO mice exhibit a strong decrease in GSIS (61). Whether ER redox is compromised in *Txnrd1*-KO β cells is not known, but upregulation of compensatory antioxidant pathways was reported, including glutathione synthesis, that was not observed with shRNA knockdown or pharmacological inhibition (61). Importantly, glutathione can also be used as an ER redox buffer and may be the primary ER reductant in some cell types, such as hepatocytes (37); however, in β cells, glutathione may not be optimal for the demands of proinsulin folding. Future studies exploring the ability of individual redox carriers to counter oxidizing stresses may provide additional insight into how β cell ER redox poise is maintained in response to stress.

In T2D, the deterioration of β cell function is a defining feature of the transition from insulin resistance to overt diabetes (62, 63). The cellular mechanisms leading to β cell dysfunction are not completely understood, yet studies from human and rodent β cells consistently demonstrate 2 primary defects. First, mitochondrial dysfunction directly impairs glucose sensing and exocytic coupling and contributes to oxidant stress (41, 64–66). Second, defects in the β cell’s insulin biosynthetic pathway lead to impaired proinsulin maturation and decreased insulin granule stores despite continued proinsulin synthesis (15, 67–69). Data from the current study suggest that the decline in metabolic function and the insulin granule deficit are linked via dysregulation of ER redox homeostasis and impaired proinsulin trafficking. We show that loss of mitochondrial function elicits ER hyperoxidation and limits ER proinsulin export for insulin granule biosynthesis. In the hyperoxidized ER environment, proinsulin disulfide bonds can readily form; however, resolution of non-native disulfides from mispaired cysteines may be severely attenuated due to insufficient reductive capacity. Furthermore, ER hyperoxidation may also interfere with ER clearance mechanisms. ER-associated degradation relies on ERdj5-mediated reduction of terminally misfolded proteins for retrotranslocation and subsequent proteasomal degradation (70). Thus, misfolded proteins containing non-native disulfides may accumulate in a hyperoxidized ER environment. In support of this, studies from human T2D β cells and rodent diabetes models show increased levels of misfolded proinsulin in the ER as non-native monomers that form high–molecular weight, disulfide-linked proinsulin oligomers (20, 30, 31). Interestingly, Golgi alterations, including dilated cisternae, have also been reported in diabetes models accompanied by Golgi exit delays (48). Whether Golgi changes are related to proinsulin misfolding or other defects in ER function is not clear but potentially could arise from misfolded proinsulin aggregates that escaped ER quality control.

Metabolic dysfunction in the β cell may lead to ER hyperoxidation via several mechanisms that converge on limiting the available supply of cellular redox donors. First, diminishing metabolic activity can directly decrease NADPH cycling necessary to sustain thioredoxin reduction by TXNRD1. This was evident in our studies following direct suppression of glucokinase using mannoheptulose as well as KD of *Idh1* or *Txnrd1*. Second, mitochondrial dysfunction can increase production of reactive oxygen species, including superoxide radicals and hydrogen peroxide (33, 71). Thus, diversion of cellular reductants to combat oxidative stress may restrict ER access to critical redox buffers. In support of this, we previously demonstrated that hydrogen peroxide scavenging via ebselen can restore ER redox control and improve β cell function in diabetes models (33). Third, TXNIP, which is upregulated in models of β cell dysfunction and T2D (72, 73), binds and sequesters reduced thioredoxin and thereby limits thioredoxin availability for ER redox buffering (74, 75). Here, we show that *Txnip* suppression improves ER redox control, proinsulin trafficking, and β cell function. Finally, as proinsulin synthesis increases in response to insulin resistance (1, 76), the increased demand for proinsulin folding may strain the metabolic supply of reductants that buffer ER redox. When this demand exceeds the ER’s redox capacity, ER hyperoxidation occurs and defects in proinsulin folding emerge. In support of this, decreasing proinsulin synthesis via heterozygous or homozygous deletion of *Ins2* in *Ins1*-KO mice attenuates ER stress in β cell dysfunction models (77). In contrast, genetic inhibition of ER-Golgi transport in β cells elicits proinsulin misfolding (78), suggesting that relieving the ER of client proteins is critical to ER redox homeostasis.

While our data highlight the importance of the fine-tuned balance of cellular redox cycles in control of ER function and insulin biosynthesis, clinical trials using antioxidants with a primary endpoint of glucose control in diabetes (hemoglobin A1c) are limited (79–81). Current challenges to this treatment paradigm include the development of antioxidant agents with sufficient potency and specificity to attenuate oxidizing stress in β cells in vivo. Whether more specific antioxidant agents such as the *TXNIP* inhibitor, SRI-37330, will be efficacious in treating T2D remains to be determined (53, 56), but our studies encourage the continued exploration into antioxidant pathways that may be utilized to promote β cell health and function.

## Methods

### Sex as a biological variable.

Sex as a biological variable was addressed by examining male and female animals, and similar findings are reported for both sexes. Similarly, male and female human islets were utilized in studies as reported.

### Cell culture, islet isolation, and reagents.

Cell culture reagents were from Life Technologies, Thermo Fisher Scientific, unless specified otherwise. Chemical reagents were from MilliporeSigma unless specified otherwise. BSA-conjugated fatty acid solution was prepared as previously described (33). INS-1 832/3 cells (a gift from Christopher Newgard, Duke University, Durham, North Carolina, USA) were cultured as previously described (82). INS-1 832/3 cells stably expressing proCpepSNAP have been described previously (47). Cells were transduced with 1 × 10^7^ to 5 × 10^7^ infectious units/mL adenovirus for 18 hours and assayed 72–96 hours after treatment. Cells were transfected with siRNAs targeting rat BiP (*Hspa5*) or a nontargeted siRNA control using Dharmafect I (Horizon Discovery) and assayed 72 hours after transfection. Mouse islets were isolated via collagenase V digestion and purified using Histopaque 1077 and 1119. Islets were cultured in RPMI supplemented with 10% fetal bovine serum and 1% penicillin and streptomycin and maintained at 37°C in 5% CO_2_. Human islets obtained from Alberta Diabetes Institute IsletCore were cultured in CMRL supplemented with 10% fetal bovine serum and 1% penicillin and streptomycin. Pools of islets were transduced with 1 × 10^8^ to 5 × 10^8^ infectious units/mL adenovirus for 18 hours and assayed 72–96 hours after treatment.

### Animal studies.

The generation of *Clec16a^fl/fl^ Ins1^tm1.1(cre)Thor^* mice on C57BL6/N background has been described previously (42, 44, 83). The *Ins1^tm1.1(cre)Thor^* allele was maintained as hemizygous, and no difference in glucose tolerance, GSIS, or insulin content has been observed compared to wild-type mice (45). C57BL6/J mice (Jackson Laboratory) were bred in-house and equally represent male and female mice in all studies. Hyperglycemia (blood glucose > 180 mg/dL) was confirmed in β cell *Clec16a*-KO animals prior to the experiment, which were compared with age-matched, normoglycemic littermate controls. Blood glucose was determined using a One Touch Ultra 2 glucometer. Plasma insulin was determined by ELISA (ALPCO).

### Plasmids and viruses.

Generation of the following adenoviruses has been described previously: AdRIP-ERroGFP (33), AdRIP-proCpepSNAP (47), Ad-shIdh1 (50), Ad-shSAFE (50), and AdRIP-proCpepRUSH (48). AdRIP-Grx1-GFP2 was a gift from Amelia Linneman (Indiana University School of Medicine, Indianapolis, Indiana, USA) (40). The NADPH sensor, iNAP1, containing NADP(H)-binding domains of T-Rex ligand binding pocket, and the iNAPc (control, NADPH insensitive) were gifts from Yi Yang, East China University of Science and Technology, Shanghai, China (39). iNAP1 and iNAPc were subcloned into pENTR2b-RIP. Primers containing Txnrd1 shRNA target sequences were inserted into pU6-MCS-PGK-mCherry by restriction digest (50). Shuttle vector cassettes were transferred into a modified pAd-PL/DEST backbone via Gateway cloning using LR Clonase II. Recombinant adenoviruses were generated in HEK293 cells (Thermo Fisher Scientific) and purified by cesium chloride gradient. All sequences were verified by the Iowa Institute of Human Genetics, University of Iowa.

### GSIS.

Insulin secretion in INS-1 832/3 cells was measured by static incubation as previously described (84). Cells were lysed in RIPA buffer, and total protein was measured by BCA assay (Pierce, Thermo Fisher Scientific). Isolated mouse islets and human islets were assayed for insulin secretion by static incubations performed on pools of 10 islets as previously described (50). Perifusion was performed using a BioRep Perifusion system with a flow rate of 100 μL/min at 37°C using pools of 40 islets as previously described (50) with the following modifications. Following stabilization at 1 mM Glc (basal) for 32 minutes, islets were stimulated with 2.5 mM, 5.6 mM, 12 mM, 16.7 mM, and 20 mM Glc for 10 minutes each. Perifusate was collected every 2 minutes under basal conditions and every minute under stimulatory conditions. Islets were lysed in RIPA for insulin content quantification. Insulin levels were measured by ELISA (rodent 80-INSMR-CH10; ALPCO). Proinsulin content was measured by ELISA (rat/mouse 10-1232-01; Mercodia), which reports no cross-reactivity with insulin or C-peptide.

### RT-qPCR.

RNA from INS-1 832/3 cells was harvested using a Zymo RNA minikit. RNA from mouse islets was harvested using the RNeasy Micro Kit (QIAGEN). cDNA was synthesized using iScript (Bio-Rad). qPCR reactions were performed using QuantStudio-7 FLEX or QuantStudio-7 PRO detection system and Design and Analysis Software (Applied Biosystems, Thermo Fisher Scientific). Expression analysis was compared to either cyclophilin B (*Ppib*) or hypoxanthine-guanine phosphoribosyltransferase (*Hprt*).

### Fluorescence microscopy and imaging.

INS-1 832/3 cells were plated on HTB9-coated coverslips as previously described (47, 84, 85). Isolated mouse islets were dispersed into monolayers using Accutase (MilliporeSigma) and plated onto HTB9-coated coverslips as previously described (47, 84, 85). ProCpepSNAP pulse-chase labeling (33, 47) and proCpepRUSH trafficking (48) were performed as previously described. Human islets were dispersed into monolayers 24 hours after arrival using Accutase and plated onto collagen-coated coverslips as described (86).

For immunostaining, cells were incubated overnight with antibodies raised against BiP (rabbit; gift of Christopher Nicchitta, Duke University, Durham, North Carolina, USA; 1:200), GRASP55 (rabbit, Proteintech 10598-AP, 1:500), GM130 (mouse, BD Transduction 610-823, 1:200), TGN38 (mouse, Novus Biologicals, Bio-Techne, NB300-575, 1:200), or proinsulin (mouse, MyBioSource MBS660187, 1:200) as indicated. Highly cross-adsorbed fluorescence-conjugated secondary antibodies were used for detection: whole IgG, donkey anti-rabbit rhodamine red-X (Jackson ImmunoResearch 711-295-152), or donkey anti-mouse Alexa Fluor 647 (Jackson ImmunoResearch 715-606-150). Cells were counterstained with DAPI and mounted using Fluorosave (Calbiochem). Images were captured on a Leica SP8 confocal microscope using an HC PL APO CS2 63×/1.40 oil objective with 3× zoom as *Z*-stacks (5 per set, 0.3 μm step, 0.88 μm optical section) and deconvolved (Huygen’s Professional).

The ratio of proCpepSNAP localization between the Golgi and ER was determined as previously described (33). Briefly, the fluorescence intensity of proCpepSNAP within the Golgi is measured using a region of interest mask defined by TGN38 or GM130 staining. This masked region is generated by a user-defined threshold for staining to eliminate background (and held constant across all images for a given study). Following determination of the proCpepSNAP-Golgi staining intensity, the Golgi region is cleared from the proCpepSNAP channel for subsequent analysis of ER localization. The intensity of proCpepSNAP staining within the ER is measured using a region of interest mask defined by BiP staining. The proCpepSNAP localization between the Golgi and ER is determined from the ratio of Golgi and ER proCpepSNAP intensity. Proinsulin localization was similarly determined but defined from Golgi or non-Golgi staining.

Granule distance measurements from the Golgi were determined using a distance transformation module in Imaris (Bitplane) from spot-rendered proCpepRUSH-positive or proCpepSNAP-positive granules (150–300 nm) and surface rendering of the Golgi identified through GM130 immunostaining. Granule distances were binned as indicated and expressed as a percentage of the total to normalize between cells and conditions (33, 47, 48).

For redox measurements (ER-roGFP, iNAP1, Grx1-roGFP), cells were imaged on an inverted Olympus IX83 microscope with a 20× objective (HC PL APO CS2; 0.75 NA) using Chroma 49 002 GFP/CY2 Bandpass (470/535 nm) and custom Chroma BX3-mounted (395/510 nm) filters. To examine glucose-dependent changes in redox cycles, cells were cultured in secretion assay buffer (82) containing 2 mM glucose for 20 minutes, imaged, treated with 20 mM glucose for 12 minutes, and reimaged. Subsequently, cells were sequentially treated with 10 mM DTT followed by 5 mM diamide for 12 minutes each and imaged to establish fully reduced or oxidized states of the reporter for normalization, respectively. Fluorescence intensities of each channel were calculated from masked images (to remove background) of whole cells using macros written for Fiji NIH software. Ratiometric intensities (395/470 nm) were normalized by comparing to DTT- and diamide-treated samples using GraphPad Prism software.

### Ultrastructure.

Isolated islets were fixed in 2.5% glutaraldehyde, 4% formaldehyde cacodylate, buffer overnight (16–18 hours) at 4°C. Tissue was postfixed in fresh 1% OsO_4_ for 1 hour, dehydrated using a graded alcohol series followed by propylene oxide, and embedded in Epon resin. Resin blocks were trimmed with a glass knife, cut to ultrathin (50–70 nm) sections with a diamond knife, and mounted on Formvar-coated copper grids. Grids were double contrasted with 2% uranyl acetate, then with lead citrate. Images were captured at 4,000× original magnification by a Hitachi HT7800 transmission electron microscope. We quantified β cell area and insulin granules manually using ImageJ software (NIH, 7 images per sample). Mature and immature granules were distinguished based on core density and the presence of the characteristic halo for mature granules.

### Mitochondrial activity measurements.

INS-1 832/3 cells were seeded at a density of 40,000 cells/well in Agilent Seahorse XF 96-well microplates in triplicate. Cells were cultured for 72 hours in BSA (control) or OPG medium as indicated. Prior to the assay, cells were washed and incubated in serum-free DMEM supplemented with 5 mM HEPES, 2 mM l-glutamine, and 1 mM Na pyruvate (Life Technologies, Thermo Fisher Scientific), pH 7.4, with 20 mM glucose. Cellular respiration was evaluated by monitoring the oxygen consumption rate using the Agilent Seahorse XF96 Extracellular Flux Analyzer. A basal rate was established, followed by addition of 2.5 μM oligomycin A, 1.25 μM FCCP, and 2 μM rotenone and antimycin, each as sequential injections. Data were normalized following postexperiment cell enumeration.

Isolated mouse islets were seeded at 100 islets per well in duplicate into Agilent Seahorse islet capture plates containing Agilent Seahorse XF assay media supplemented with 1% fetal bovine serum, 2 mM glutamine, and 2 mM glucose. Oxygen consumption was measured using the Agilent Seahorse XFe24 machine at 2.5 mM glucose (3 cycles) followed by injections of 16.7 mM glucose (6 cycles), 5 μM oligomycin (8 cycles), and 5 μM each of rotenone and antimycin A (8 cycles). Data were normalized to islet number.

For indirect measures of cellular respiration, INS-1 832/3 cells were seeded in a 96-well plate at 10^4^ cells/well in culture medium in triplicate. Following treatment, CCK8 solution (GlpBio) was added per manufacturer instruction, and absorbance was measured at 450 nm following 2 hours of incubation.

### Statistics.

Data are presented as the mean ± SEM. For statistical significance determinations, data were analyzed by the 2-tailed unpaired Student’s *t* test or by 1-way or 2-way ANOVA with post hoc analysis for multiple-group comparisons as indicated (GraphPad Prism). A *P* < 0.05 was considered significant.

### Study approval.

Animal protocols were approved by and performed in accordance with the University of Iowa’s Institutional Animal Care and Use Committee.

### Data availability.

All primer sequences are provided as Supplemental Table 1. Human donor information is provided as Supplemental Table 2. Supporting Data Values are provided for figures.

## Author contributions

KER and SBS conceived and designed studies. KER, NJS, GLP, EMW, and SBS performed the experiments and analyzed the data. SAS created and provided the *Clec16a* mouse line. KER and SBS wrote the manuscript.

## Supplementary Material

Supplemental data

Unedited blot and gel images

Supplemental table 1

Supplemental table 2

Supporting data values

## Version 1 06/27/2024

In-Press Preview

## Version 2 08/08/2024

Electronic publication
