# Supplementary material for: A metabolic redox relay supports ER proinsulin export in pancreatic islet β cells
Source: JCI Insight. 2024 Jun 27;9(15):e178725. doi: 10.1172/jci.insight.178725 (PMC11383593; doi:10.1172/jci.insight.178725)

A metabolic redox relay supports ER proinsulin export in pancreatic islet β-cells

Kristen E. Rohli[1,2,3], Nicole J. Stubbe[1], Emily M. Walker[4], Gemma L. Pearson[4], Scott A. Soleimanpour[4,5,6], and Samuel B. Stephens[1,2,3,7]

[1]Fraternal Order of Eagles Diabetes Research Center, University of Iowa, Iowa City, IA, USA
[2]Interdisciplinary Graduate Program in Genetics, University of Iowa, Iowa City, IA, USA
[3]Department of Internal Medicine, Division of Endocrinology and Metabolism, University of Iowa, Iowa City, IA, USA
[4]Division of Metabolism, Endocrinology & Diabetes and Department of Internal Medicine, University of Michigan, Ann Arbor, MI, USA
[5]Department of Molecular and Integrative Physiology, University of Michigan, Ann Arbor, MI, USA
[6]VA Ann Arbor Healthcare System, Ann Arbor, MI, USA.

[7]Corresponding author:
Samuel B. Stephens, Ph.D.
Fraternal Order of Eagles Diabetes Research Center
Department of Internal Medicine, Division of Endocrinology and Metabolism
University of Iowa
Iowa City, IA 52246
Email: samuel-b-stephens@uiowa.edu
Tel. 319-335-4843

Running title: Metabolic regulation of ER redox homeostasis
Key words: endoplasmic reticulum, thioredoxin, NADPH, mitochondria, β-cell function

Supplemental figure legends

Supplemental Figure 1. β-cell dysfunction coincides with impaired NADPH and GSH flux. (**A**-**C** and **G-J**) INS-1 832/3 cells were cultured for 72 h in control media containing BSA or media with oleate: palmitate (2:1, 1 mM) and elevated glucose (20 mM) termed OPG as indicated. Insulin content (**A**), proinsulin content (**B**), and the proinsulin/insulin ratio (**C**) were measured from whole cell lysates. (**D**-**F**) INS-1 832/3 cells were treated for 4 h with MG132 (10 μM). (**D**) ER redox was measured via ratiometric imaging ERroGFP (AdRIP) (n=5). Cell lysates were analyzed by immunoblot for poly-ubiquitinated proteins **(E**) and re-probed for γ-tubulin (**F**). (**G**) INS-1 832/3 cells were cultured for 3 d in BSA or OPG media, or OPG media for 3 d followed by BSA for an additional 3 d. ER redox was measured via ratiometric imaging ERroGFP (AdRIP) (n=5). NADPH/$NADP^+$ (**H** and **I**) or GSSG/GSH (**J**) were measured by sequential incubation in 2 mM Glc followed by 20 mM Glc for 12 min each via ratiometric imaging iNAP (n=4), iNAPc (n=4), or Grx1-roGFP (n=7-8) (AdRIP), respectively. Normalized data are presented in Figure 1, H and I. Data represent the mean ± S.E.M. * $p < 0.05$ or not significant (ns) by Student t test (**A**-**D**), one-way ANOVA with Tukey post-test (**G**), or two-way ANOVA with Tukey post-test analysis (**H**-**J**).

Supplemental Figure 2. Impaired β-cell function in male and female *Clec16a* KO mice. *Clec16a^fl/fl^* (WT) and *Clec16a^fl/fl^; Ins1-Cre* (*Clec16a* KO) mice were used as follows. (**A**) Ad lib fed blood glucose was monitored weekly in female mice beginning at 4 wks old (n=7-9). (**B**) Plasma insulin was measured from 4 h fasted 14 wk old female mice (n=4). (**C**) Body weight was monitored in female mice beginning at 4 wks old (n=7-9 per group). (**D**) Insulin secretion was measured in islets from 14 wk old female mice by static incubation at 2.5 mM Glc followed

by 12 mM Glc for 1 h each (n=9). (**E**) Islet insulin secretion from 13-15 wk old male mice was assayed by perifusion using a glucose ramp as indicated (n=5). NADPH/NADP$^+$ (**F**, n=10-11) and GSSG/GSH (**G**, n=5-6) were measured in islets from 14-20 wk old male and female mice by sequential incubation in 2 mM Glc followed by 20 mM Glc for 12 min each via ratiometric imaging iNAP or Grx1-roGFP2 (AdRIP), respectively. Normalized data are presented in Figure 2, G and H. (**H**) mRNA expression was measured by RT-qPCR in islets from 18 wk old male and female mice (n=4-9). (**A-H**) Data represent the mean ± S.E.M. * $p < 0.05$ or not significant (ns) by two-way ANOVA with mixed-model post-test analysis (**A**, **C**, and **E**), Student t test (**B**), two-way ANOVA with Sidak post-test analysis (**D**), two-way ANOVA with Tukey post-test analysis (**F** and **G**), or multiple t test (**H**).

Supplemental Figure 3. β-cell *Clec16a* KO decreases insulin, but not proinsulin content, and impairs insulin granule formation. *Clec16a$^{fl/fl}$* (WT) and *Clec16a$^{fl/fl}$; Ins1-Cre* (*Clec16a* KO) mice were used as follows. Insulin (**A**) and proinsulin (**B**) content were determined from islet cell lysates from 14-18 wk old female mice (n=9 or 6, respectively). Data represent the mean ± S.E.M. * $p < 0.05$ or not significant (ns) by Student t test.

Supplemental Figure 4. Verification of BiP antibody. INS-1 832/3 cells were transfected with non-targeting control siRNA (control) or 2 distinct siRNA duplexes targeting BiP (#1 or #2, respectively). (**A**) mRNA expression of BiP was determined by RT-qPCR (n=4). (**B** and **C**) Cell lysates were examined by immunoblot. BiP expression was quantified normalized to γ-tubulin (**B**; n=4) with a representative blot shown (**C**). (**D**) Fixed cells were immunostained for BiP (red) and counterstained with DAPI (blue). Representative images are shown. (**A** and **B**) Data

represent the mean ± S.E.M. * $p < 0.05$ or not significant (ns) by one-way ANOVA with Dunnett post-test analysis.

Supplemental Figure 5. Quantification of proCpepSNAP localization in β-cell *Clec16a* KOs. (**A**) Representative workflow for image quantification of proCpepSNAP localization presented in Figure 4B. Using a region of interest mask derived from the GM130 staining (magenta), the proCpepSNAP (green) contained within the Golgi (Golgi-masked proCpepSNAP) was determined. ProCpepSNAP within the ER region was determined from the Golgi excluded images (non-Golgi masked proCpepSNAP) using a region of interest mask derived from the BiP staining (red). Scale bar = 5 μm. (**B**) Total fluorescence intensity of proCpepRUSH was quantified from data presented in Figure 4D. Data represent the mean ± S.E.M. * $p < 0.05$ or not significant (ns) by Student t test.

Supplemental Figure 6. Mannoheptulose impairs cellular redox flux in INS-1 832/3 cells and mouse islets. INS-1 832/3 cells (**A** and **C**) or 12-16 wk old male and female mouse islets (**B**) were treated with vehicle (veh), mannoheptulose (MnH, 1 mM or 2 mM, respectively), or rotenone (Rot, 2 μM) for 4 h as indicated. (**A**) Absorbance (450 nm) was measured following 2 h incubation with CCK8 reagent (n=4). NADPH/$NADP^+$ (**B**, n=4) and GSSG/GSH (**C**, n=4) were measured by sequential incubation in 2 mM Glc followed by 20 mM Glc for 12 min each via ratiometric imaging iNAP or Grx1-roGFP2 (AdRIP), respectively. Normalized data are presented in Figure 5, A and B. (**A**-**C**) Data represent the mean ± S.E.M. * $p < 0.05$ by one-way ANOVA with Dunnett's multiple comparison test (**A**), two-way ANOVA with Tukey post-test analysis (**B** and **C**).

Supplemental Figure 7. Mannoheptulose impairs proinsulin trafficking in INS-1 832/3 cells. (**A**) INS-1 832/3 cells were treated with vehicle (veh), mannoheptulose (MnH, 1 mM) for 4 h as indicated. ER redox was measured (n=4) via ratiometric imaging ERroGFP (AdRIP). (**B**) Total fluorescence intensity of proCpepSNAP labeling was quantified from data presented in Figure 5, D and E. (**C**) INS-1 832/3 cells stably expressing proCpepSNAP cells were cultured with BSA vs. OPG for 72 h or treated with MnH for 4 h as indicated. Cells were pulse-labeled with SNAP-505, chased for 10 min, and immunostained with TGN38 and BiP. The ratio of proCpepSNAP fluorescence in the Golgi (TGN38) vs. ER (BiP) was quantified (n=4).
(**D**) Representative workflow for image quantification of proCpepSNAP localization presented in Figure 5, D and E. Using a region of interest mask derived from the TGN38 staining (magenta), proCpepSNAP (green) contained within the Golgi (Golgi-masked proCpepSNAP) was determined. ProCpepSNAP within the ER region was determined from the Golgi excluded images (non-Golgi proCpepSNAP) using a region of interest mask derived from the BiP staining (red). (**A**-**C**) Data represent the mean ± S.E.M. * $p < 0.05$ by Student t test (**A**), one-way ANOVA with Dunnett's multiple comparison test (**B**), or one-way ANOVA with Tukey post-test analysis (**C**). Scale bar = 3 μm.

Supplemental Figure 8. Knockdown of *Idh1* impairs cellular redox flux in mouse islets. 12-16 wk old male and female mouse islets were treated with Ad-shSAFE or Ad-sh*Idh1* as indicated. NADPH/NADP$^+$ (**A**, n=4) and GSSG/GSH (**B**, n=5-6) were measured in mCherry+ islet cells (Ad-shRNA) by sequential incubation in 2 mM Glc followed by 20 mM Glc for 12 min each via ratiometric imaging iNAP or Grx1-roGFP2 (AdRIP), respectively. Normalized data

are presented in Figure 6, B and C. Total fluorescence intensity of proCpepSNAP was quantified from data presented in Figure 6F (**C**; n=4) and Figure 6H (**D**; n=3). (**A**-**D**) Data represent the mean ± S.E.M. * $p < 0.05$ or not significant (ns) by two-way ANOVA with Tukey post-test analysis (**A**, **B**, and **D**) or Student t test (**C**).

Supplemental Figure 9. Pharmacological inhibition of TXNRD1 impairs NADPH flux and proinsulin trafficking. (**A**) 12-16 wk old male and female mouse islets were treated with vehicle (Veh), AAPA (25 μM), or auranofin (AFN; 10 μM) for 4 h prior to imaging. NADPH/$NADP^+$ was measured in islets (n=4) by sequential incubation in 2 mM Glc followed by 20 mM Glc for 12 min each via ratiometric imaging iNAP (AdRIP). Normalized data are presented in Figure 7A. (**B**) INS-1 832/3 cells stably expressing proCpepSNAP were cultured for 72 h in BSA vs. OPG or treated for 4h with auranofin (AFN; 10 μM) as indicated. Cells were pulse-labeled with SNAP 505 (green) and chased for 10 minutes. Cells were immunostained for BiP (red) and TGN38 (magenta). Representative images are shown. The ratio of proCpepSNAP fluorescence in the Golgi (TGN38) vs. ER (BiP) was quantified and presented in Figure 7B. (**A**) Data represent the mean ± S.E.M. * $p < 0.05$ or not significant (ns) by two-way ANOVA with Tukey post-test analysis. Scale bar = 5 μm.

Supplemental Figure 10. Suppression of *Txnrd1* impairs proinsulin trafficking. Representative workflow for image quantification of proCpepSNAP localization presented in Figure 7, D and E. Using a region of interest mask derived from the GM130 staining (magenta), proCpepSNAP (green) contained within the Golgi (Golgi-masked proCpepSNAP) was

determined and compared to proCpepSNAP excluded from the Golgi (non-Golgi proCpepSNAP). Scale bar = 5 μm.

Supplemental Figure 11. Suppression of *Txnip* does not restore NADPH flux following OPG culture. (**A**) Txnip mRNA expression in islets from *Clec16a*$^{fl/fl}$ (WT) and *Clec16a*$^{fl/fl}$*; Ins1-Cre* (*Clec16a* KO) mice (18 wks old) was quantified by RT-qPCR (n=8-9). (**B** and **C**) INS-1 832/3 cells were cultured for 72 h in BSA vs. OPG with either vehicle (Veh) control or SRI-37330 (1 μM) added in the last 24 h as indicated. NADPH/NADP$^+$ was measured by sequential incubation in 2 mM Glc followed by 20 mM Glc for 12 min each via ratiometric imaging iNAP (AdRIP) (**B**) and normalized to 2 mM Glc (**C**). (**D** and **E**) Islets from 16-18 wk old female *Clec16a*$^{fl/fl}$ (WT) mice were treated with SRI-37330 (1 μM) for 24 h as indicated. (**D**) Insulin secretion was measured by static incubation in media containing 2.5 mM Glc followed by 12 mM Glc for 1 h each. (**E**) Insulin content was measured from cell lysates. (**F**) Total proCpepSNAP fluorescence intensity was measured from data presented in Figure 8, E and F. (**A-F**) Data represent the mean ± S.E.M. * $p < 0.05$ or not significant (ns) by Student t test (**A** and **E**) or two-way ANOVA with Tukey (**B**, **C**, and **F**) or Sidak (**D**) post-test analysis.

Supplemental Figure 12. *Txnip* suppression restores proinsulin trafficking. Representative workflow for image quantification of proCpepSNAP localization presented in Figure 8, E and F. Using a region of interest mask derived from the GM130 staining (magenta), the proCpepSNAP (green) contained within the Golgi (Golgi-masked proCpepSNAP) was determined. ProCpepSNAP within the ER region was determined from the Golgi excluded images (non-

Golgi proCpepSNAP) using a region of interest mask derived from the BiP staining (red). Scale bar = 5 μm.

Supplemental Figure 1

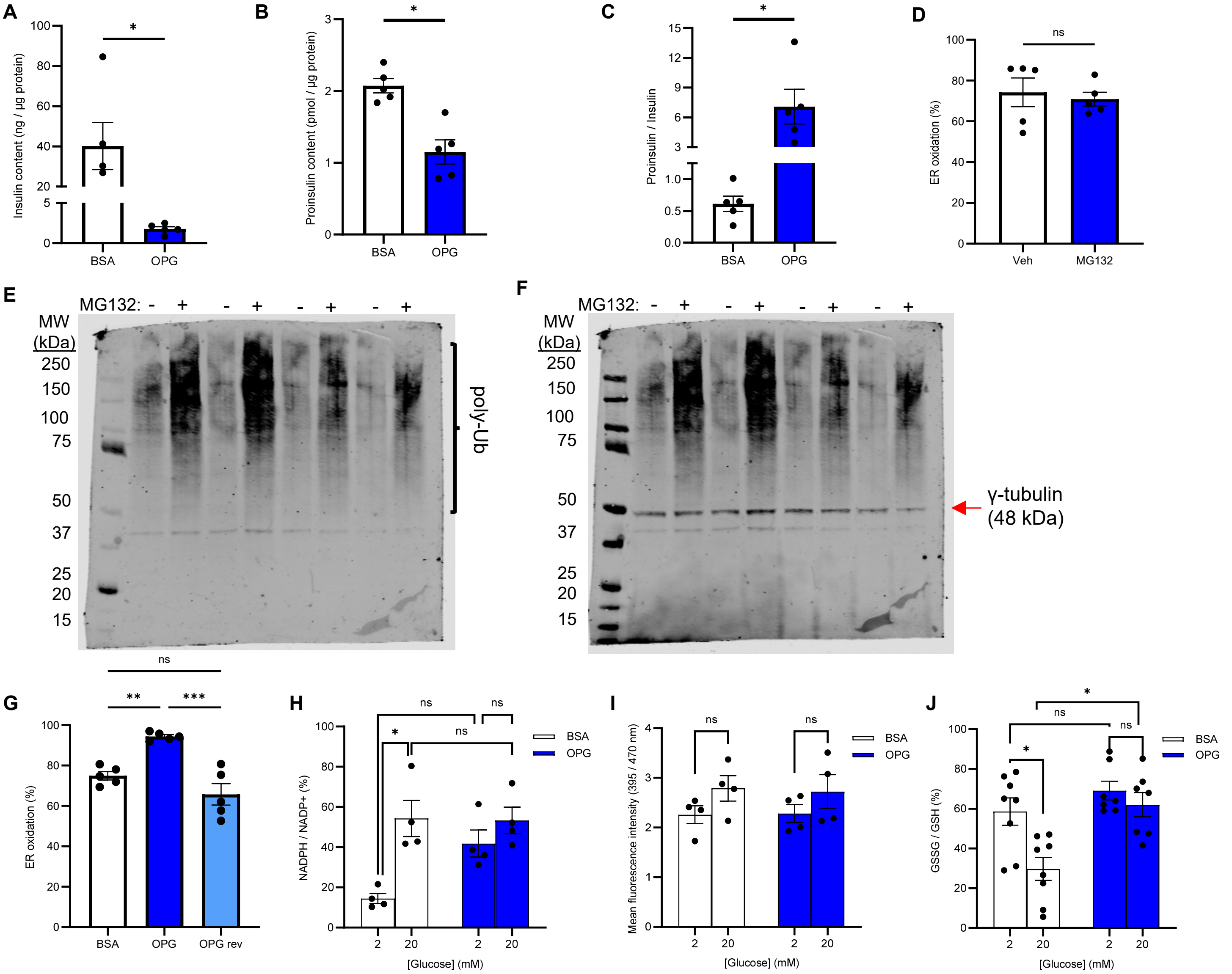

Supplemental Figure 2

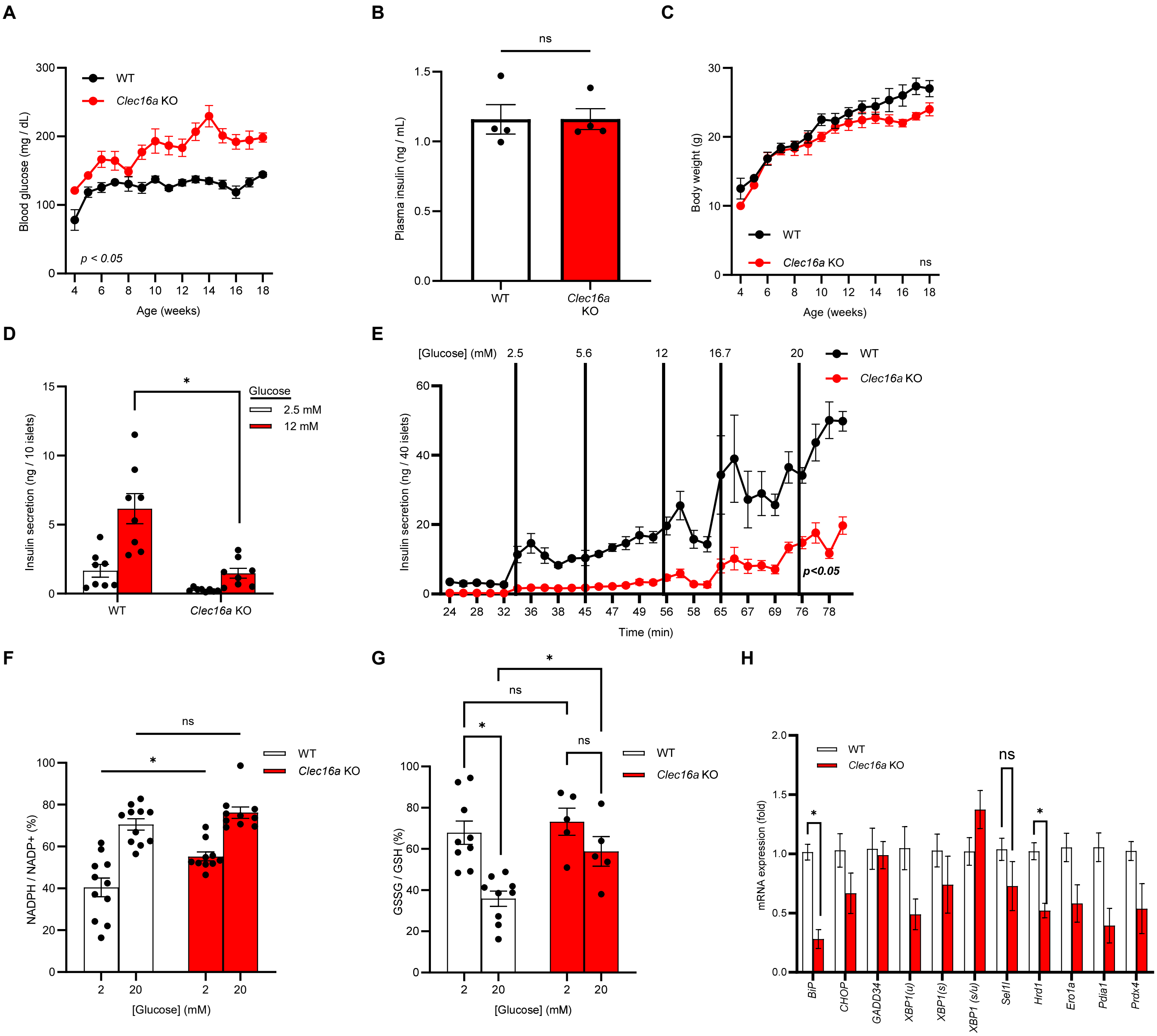

Supplemental Figure 3

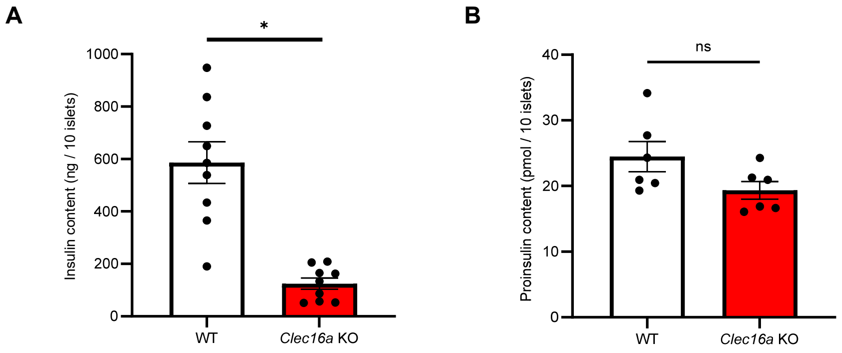

Supplemental Figure 4

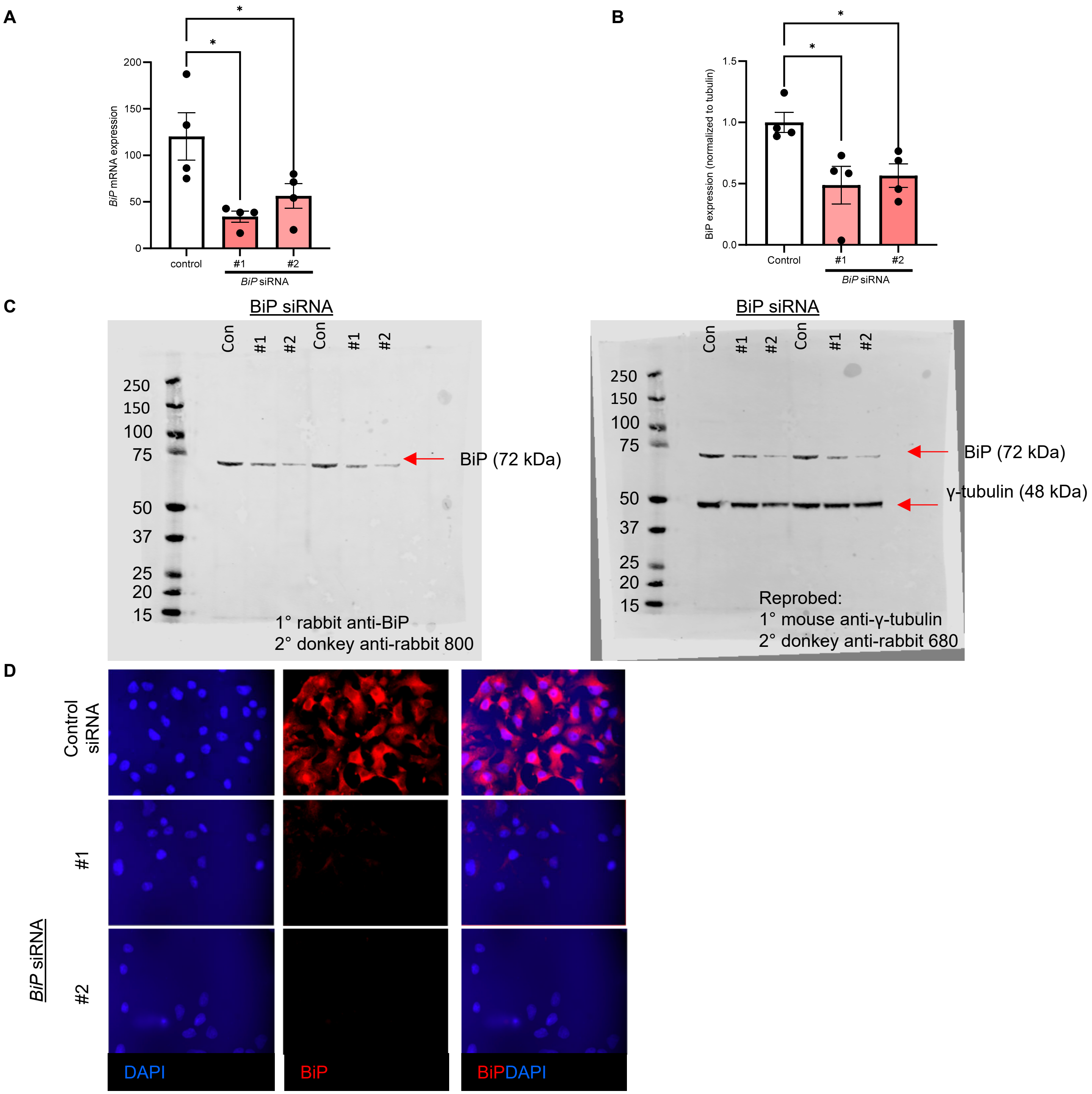

Supplemental Figure 5

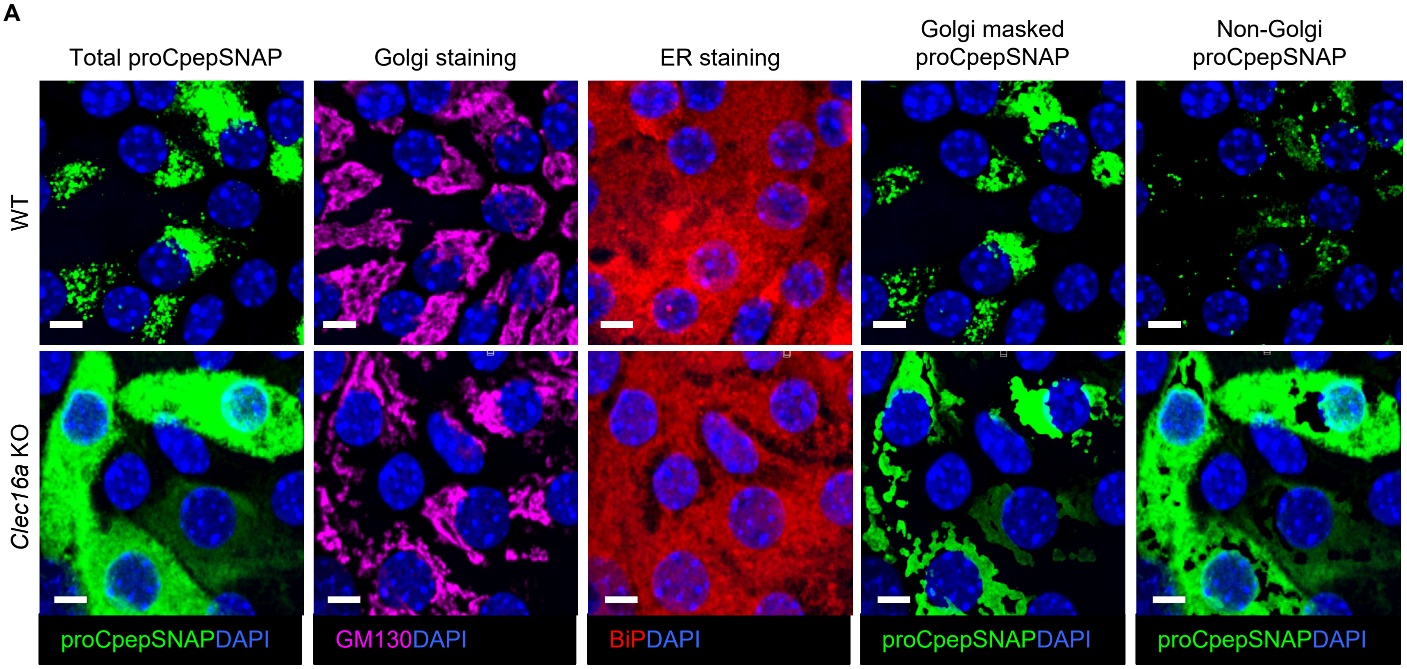

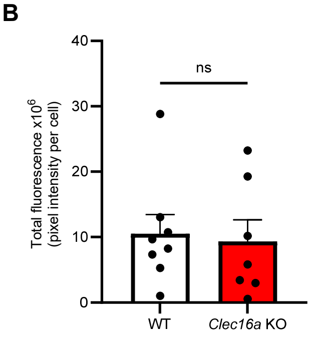

Supplemental Figure 6

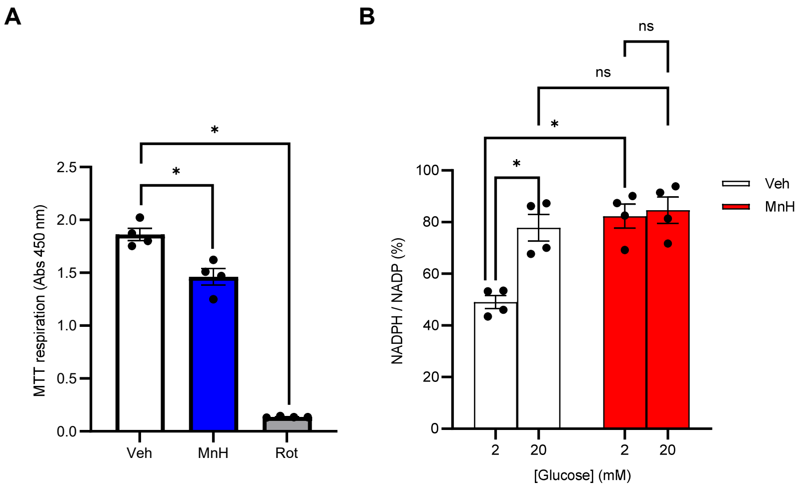

Supplemental Figure 7

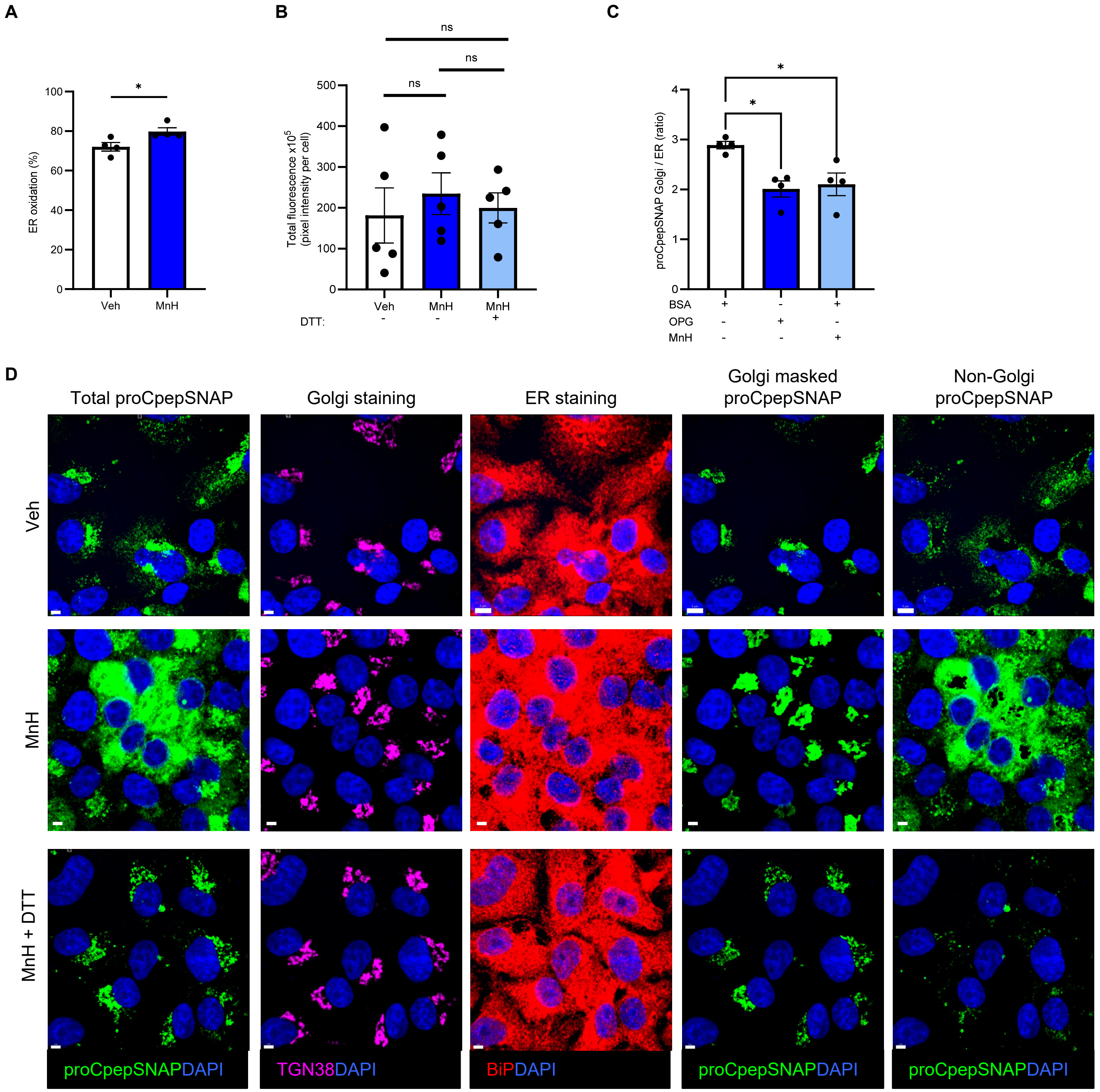

Supplemental Figure 8

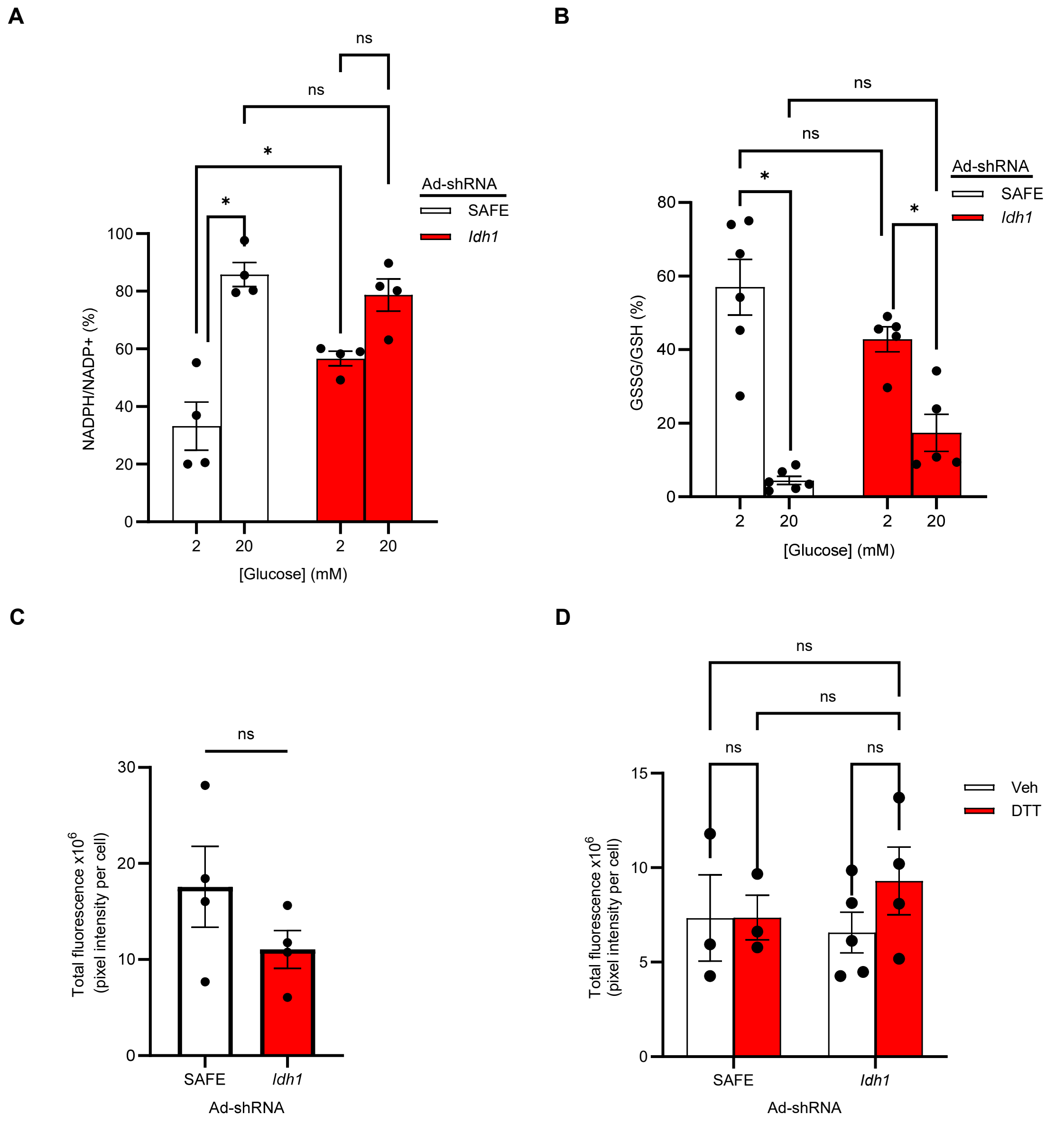

Supplemental Figure 9

A

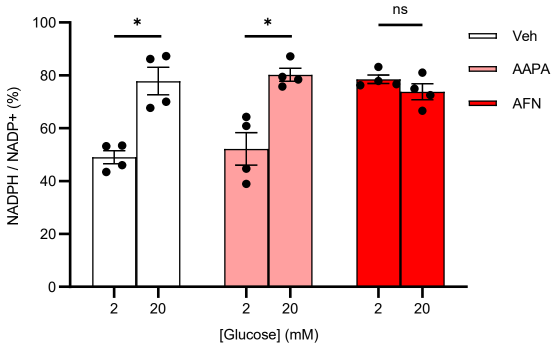

B

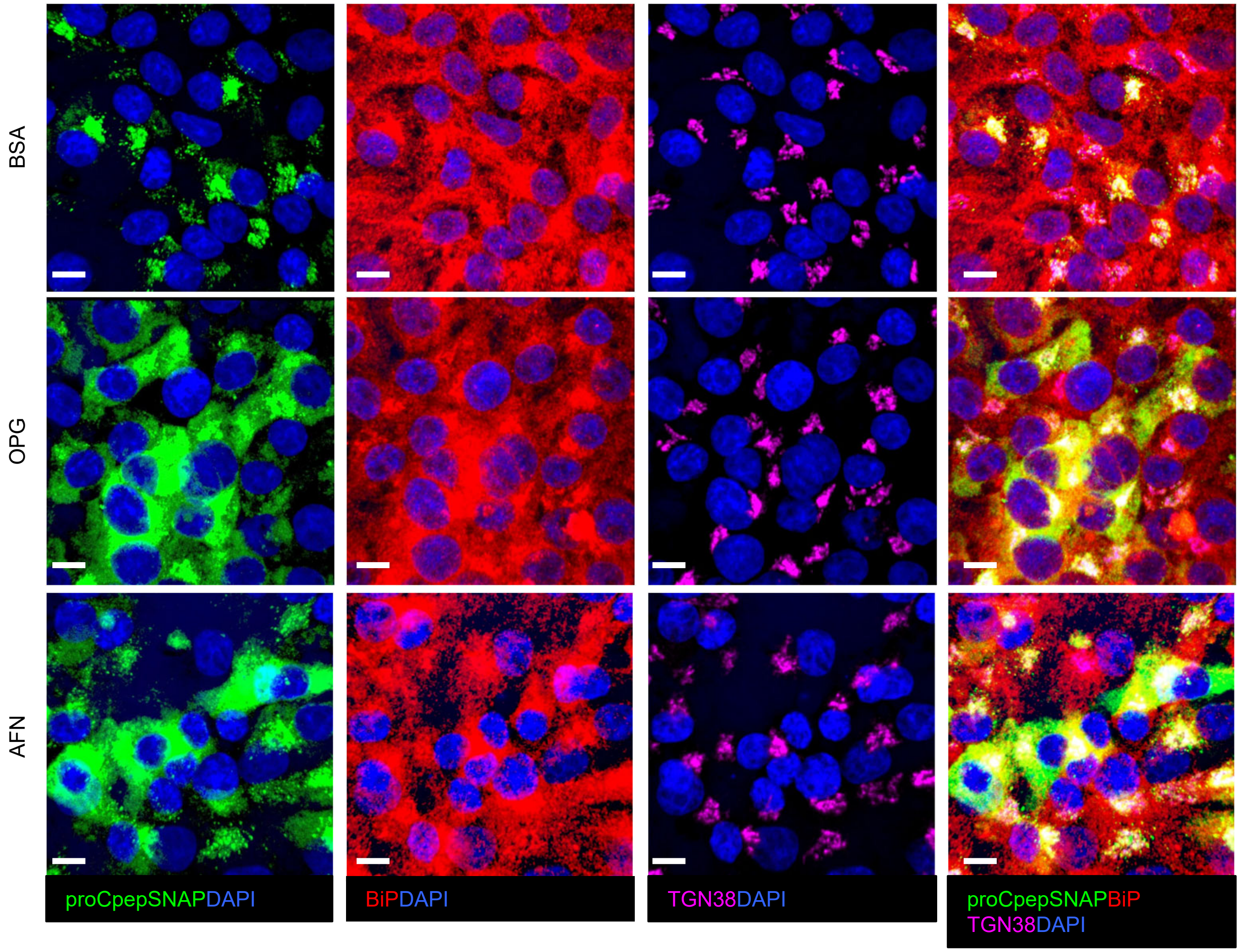

Supplemental Figure 10

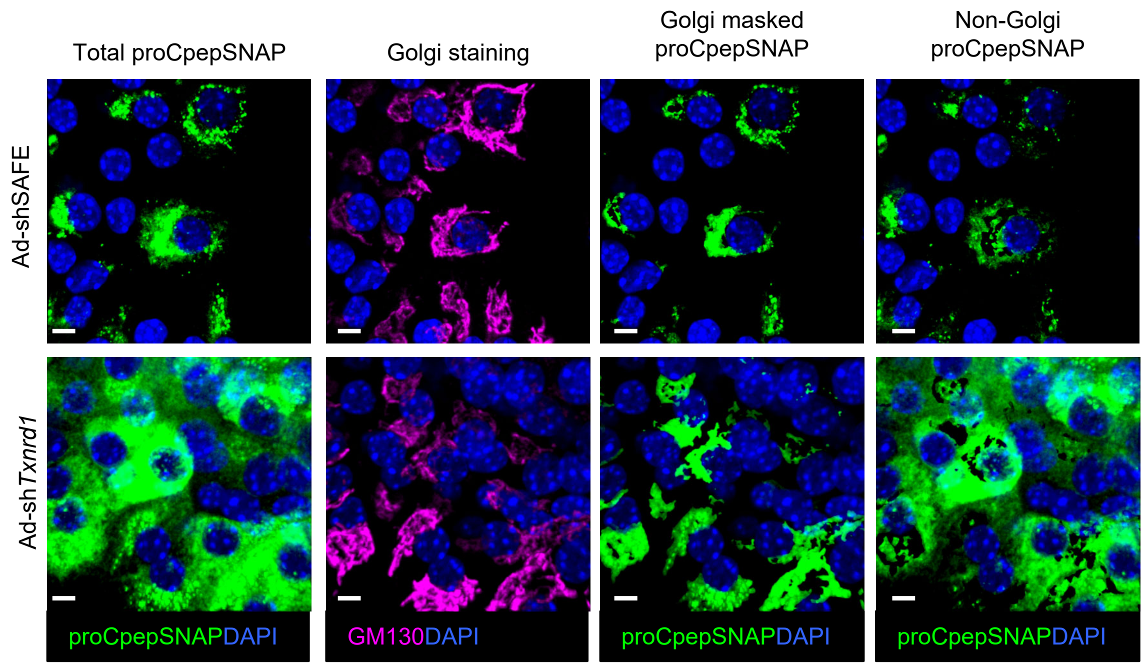

Supplemental Figure 11

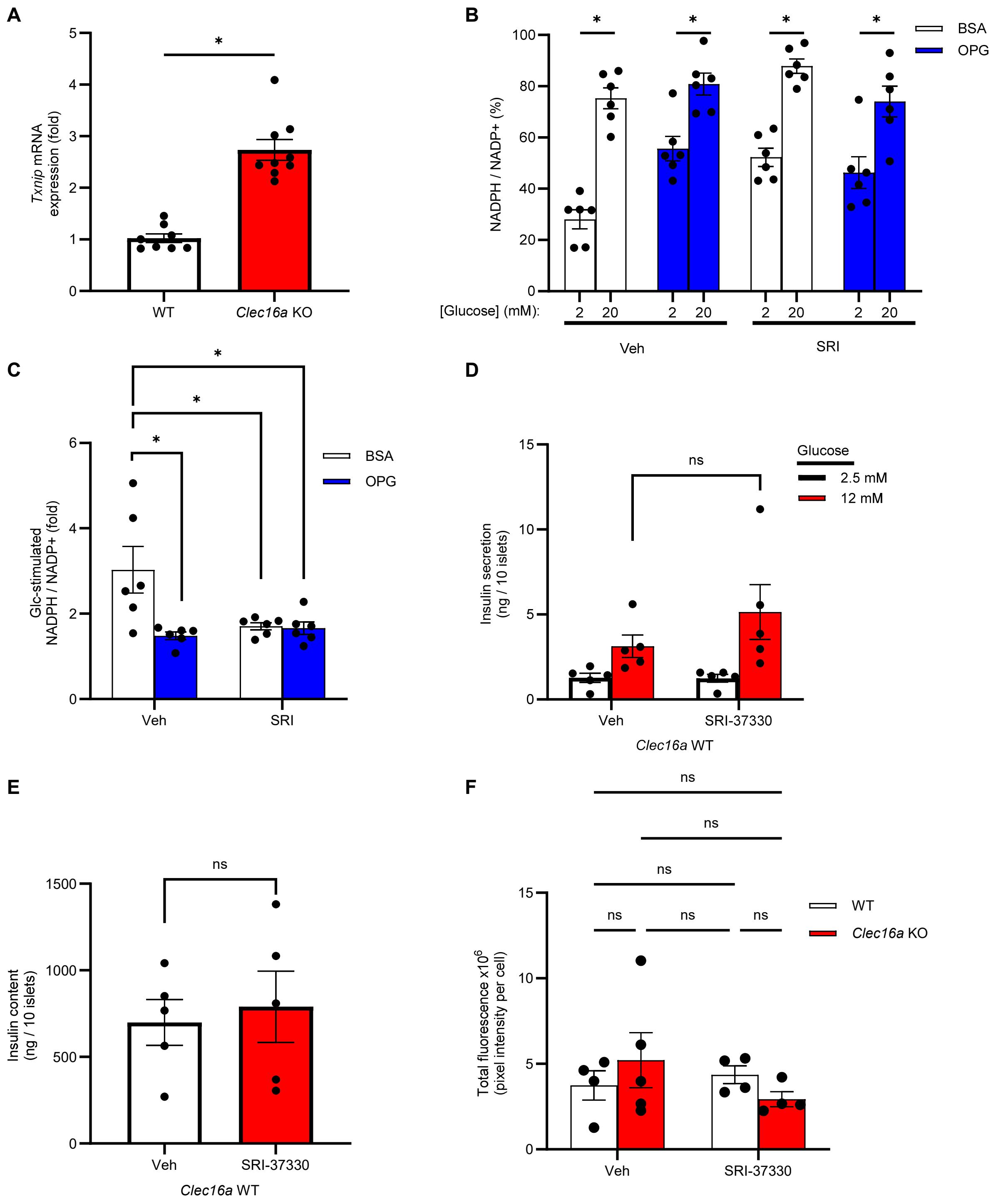

Supplemental Figure 12

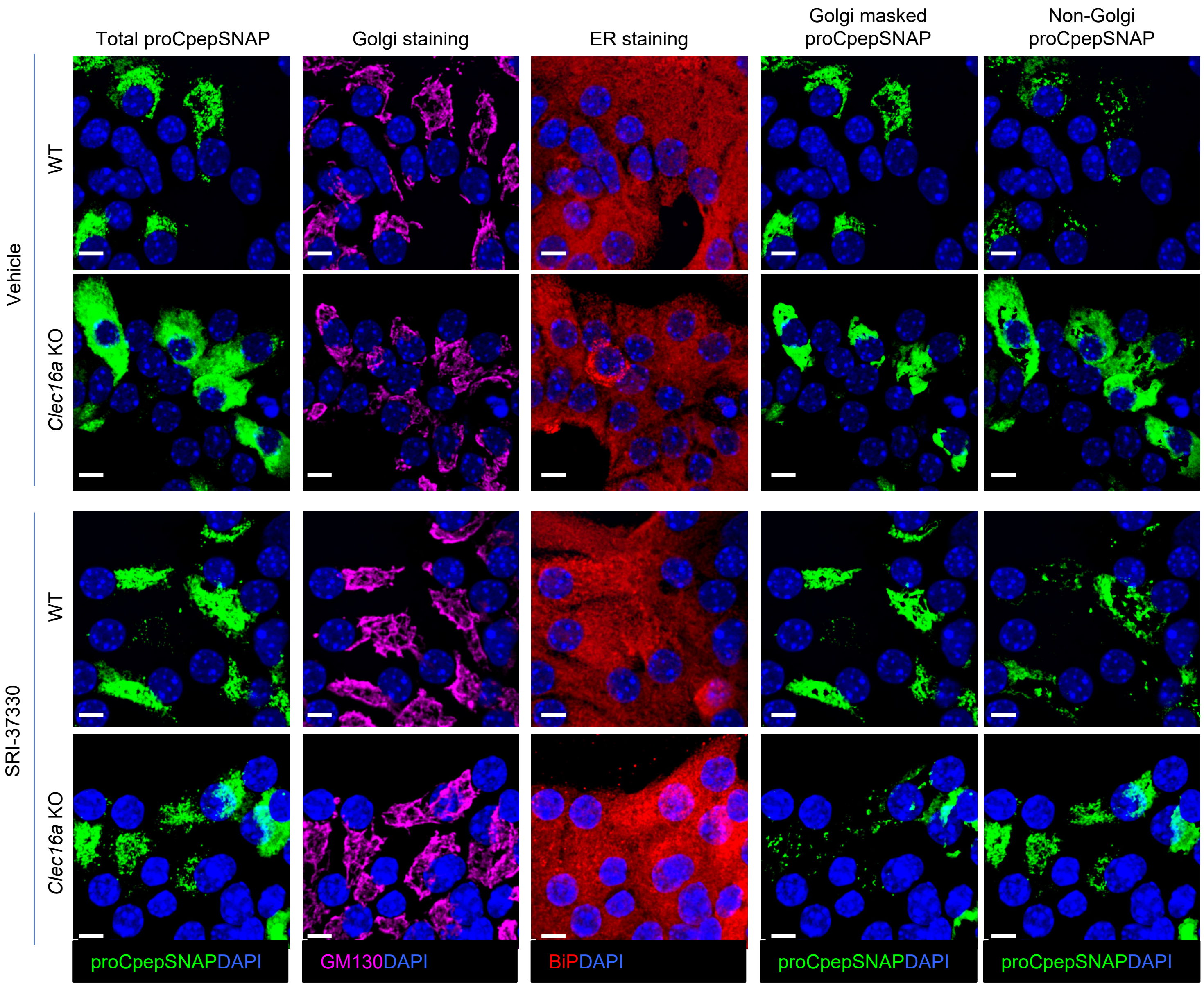

Supplement: Supplemental data [file jciinsight-9-178725-s055.pdf]
