# Supplementary material for: A metabolic redox relay supports ER proinsulin export in pancreatic islet β cells
Source: JCI Insight. 2024 Jun 27;9(15):e178725. doi: 10.1172/jci.insight.178725 (PMC11383593; doi:10.1172/jci.insight.178725)
Supplement: Unedited blot and gel images [file jciinsight-9-178725-s056.pdf]

Uncropped immunoblots used in Supplemental Figure 1

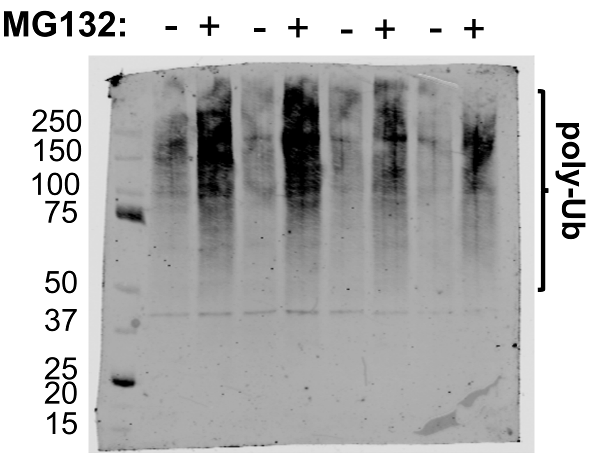

1° rabbit anti-polyubiquitin
2° donkey anti-rabbit 800

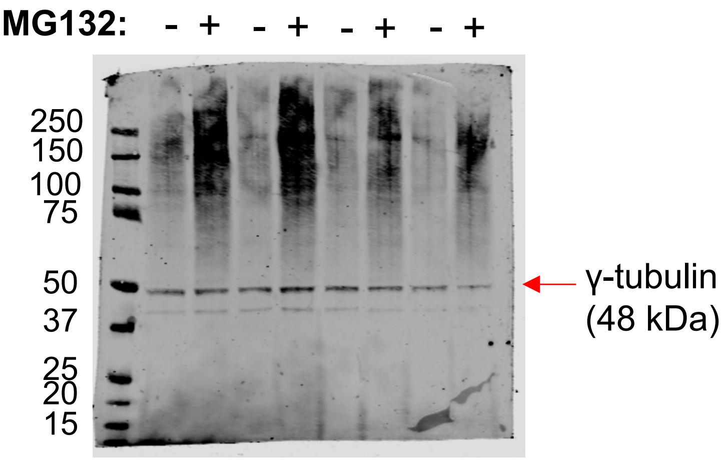

Reprobed:
1° mouse anti-γ-tubulin
2° donkey anti-rabbit 680

Uncropped immunoblots used in Supplemental Figure 4

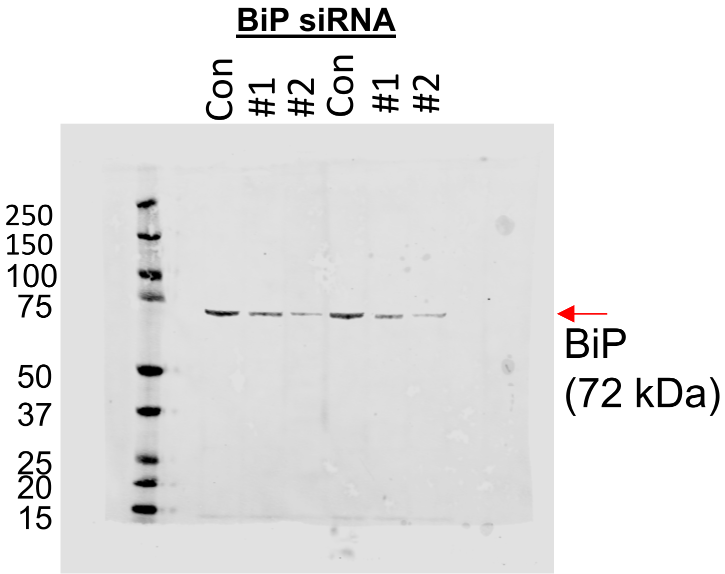

1° rabbit anti-BiP
2° donkey anti-rabbit 800

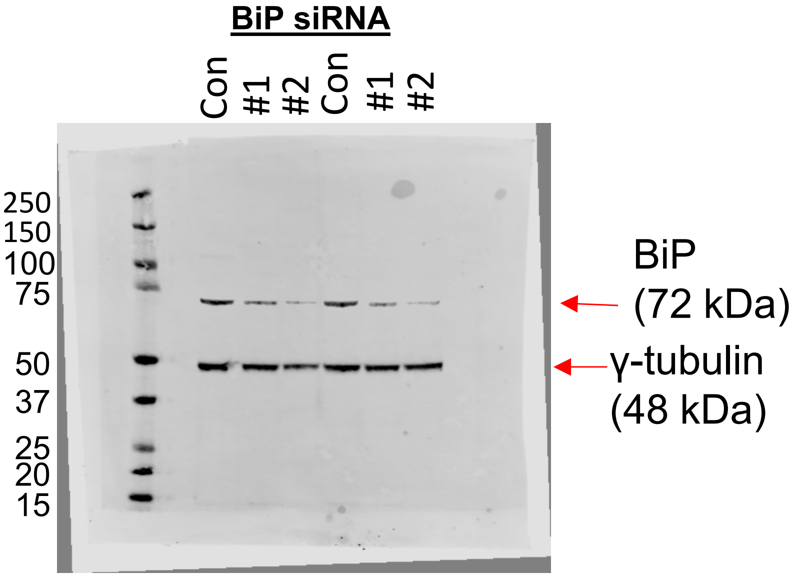

Reprobed:
1° mouse anti-γ-tubulin
2° donkey anti-rabbit 680
