# Supplementary material for: A metabolic redox relay supports ER proinsulin export in pancreatic islet β cells
Source: JCI Insight. 2024 Jun 27;9(15):e178725. doi: 10.1172/jci.insight.178725 (PMC11383593; doi:10.1172/jci.insight.178725)
Supplement: Supplemental table 2 [file jciinsight-9-178725-s058.pdf]

| Donor ID | Source | Age (years) | Sex | BMI | HbA1c (%) |
|---|---|---|---|---|---|
| R518 | Alberta Diabetes Institute IsletCore | 52 | male | 25.6 | 5.6 |
| R507 | Alberta Diabetes Institute IsletCore | 59 | female | 19.9 | 5.3 |
| R468 | Alberta Diabetes Institute IsletCore | 66 | female | 27.4 | 5.4 |
| R454 | Alberta Diabetes Institute IsletCore | 66 | male | 29.6 | 5.4 |
| R450 | Alberta Diabetes Institute IsletCore | 66 | female | 21.9 | 4.3 |
